# Evolving brain and behaviour changes in rats following repetitive subconcussive head impacts

**DOI:** 10.1093/braincomms/fcad316

**Published:** 2023-11-20

**Authors:** Wouter S Hoogenboom, Todd G Rubin, Kamalakar Ambadipudi, Min-Hui Cui, Kenny Ye, Henry Foster, Esther Elkouby, Jinyuan Liu, Craig A Branch, Michael L Lipton

**Affiliations:** The Gruss Magnetic Resonance Research Center, Albert Einstein College of Medicine, Montefiore Medical Center, Bronx, NY 10641, USA; Department of Clinical Investigation, Albert Einstein College of Medicine, Montefiore Medical Center, Bronx, NY 10641, USA; Department of Radiology, Albert Einstein College of Medicine, Montefiore Medical Center, Bronx, NY 10461, USA; Department of Neurology, Icahn School of Medicine at Mount Sinai, NewYork, NY 10029, USA; The Gruss Magnetic Resonance Research Center, Albert Einstein College of Medicine, Montefiore Medical Center, Bronx, NY 10641, USA; Department of Radiology, Albert Einstein College of Medicine, Montefiore Medical Center, Bronx, NY 10461, USA; The Gruss Magnetic Resonance Research Center, Albert Einstein College of Medicine, Montefiore Medical Center, Bronx, NY 10641, USA; Department of Radiology, Albert Einstein College of Medicine, Montefiore Medical Center, Bronx, NY 10461, USA; Department of Epidemiology & Population Health, Albert Einstein College of Medicine, Montefiore Medical Center, Bronx, NY 10461, USA; The Gruss Magnetic Resonance Research Center, Albert Einstein College of Medicine, Montefiore Medical Center, Bronx, NY 10641, USA; The Gruss Magnetic Resonance Research Center, Albert Einstein College of Medicine, Montefiore Medical Center, Bronx, NY 10641, USA; The Gruss Magnetic Resonance Research Center, Albert Einstein College of Medicine, Montefiore Medical Center, Bronx, NY 10641, USA; The Gruss Magnetic Resonance Research Center, Albert Einstein College of Medicine, Montefiore Medical Center, Bronx, NY 10641, USA; Department of Radiology, Albert Einstein College of Medicine, Montefiore Medical Center, Bronx, NY 10461, USA; Department of Physiology and Biophysics, Albert Einstein College of Medicine, Montefiore Medical Center, Bronx, NY 10461, USA; Department of Radiology, Columbia University Irving Medical Center, NewYork, NY 10032, USA; Department of Biomedical Engineering, Columbia University, NewYork, NY 10032, USA

**Keywords:** repetitive subconcussive head impacts, animal model, diffusion tensor imaging, neuropathology, behavioural assessment

## Abstract

There is growing concern that repetitive subconcussive head impacts, independent of concussion, alter brain structure and function, and may disproportionately affect the developing brain. Animal studies of repetitive subconcussive head impacts are needed to begin to characterize the pathological basis and mechanisms underlying imaging and functional effects of repetitive subconcussive head impacts seen in humans. Since repetitive subconcussive head impacts have been largely unexplored in animals, we aimed to characterize the evolution of imaging, behavioural and pathological effects of repetitive subconcussive head impacts in awake adolescent rodents. Awake male and female Sprague Dawley rats (postnatal Day 35) received 140 closed-head impacts over the course of a week. Impacted and sham-impacted animals were restrained in a plastic cone, and unrestrained control animals were included to account for effects of restraint and normal development. Animals (*n* = 43) underwent repeated diffusion tensor imaging prior to and over 1 month following the final impact. A separate cohort (*n* = 53) was assessed behaviourally for fine motor control, emotional-affective behaviour and memory at acute and chronic time points. Histological and immunohistochemical analyses, which were exploratory in nature due to smaller sample sizes, were completed at 1 month following the final impact. All animals tolerated the protocol with no overt changes in behaviour or stigmata of traumatic brain injury, such as alteration of consciousness, intracranial haemorrhage or skull fracture. We detected longitudinal, sex-dependent diffusion tensor imaging changes (fractional anisotropy and axial diffusivity decline) in corpus callosum and external capsule of repetitive subconcussive head impact animals, which diverged from both sham and control. Compared to sham animals, repetitive subconcussive head impact animals exhibited acute but transient mild motor deficits. Repetitive subconcussive head impact animals also exhibited chronic anxiety and spatial memory impairment that differed from the control animals, but these effects were not different from those seen in the sham condition. We observed trends in the data for thinning of the corpus callosum as well as regions with elevated Iba-1 in the corpus callosum and cerebral white matter among repetitive subconcussive head impact animals. While replication with larger study samples is needed, our findings suggest that subconcussive head impacts cause microstructural tissue changes in the developing rat brain, which are detectable with diffusion tensor imaging, with suggestion of correlates in tissue pathology and behaviour. The results point to potential mechanisms underpinning consequences of subconcussive head impacts that have been described in humans. The congruence of our imaging findings with human subconcussive head impacts suggests that neuroimaging could serve as a translational bridge to advance study of injury mechanisms and development of interventions.

## Teaser

Hoogenboom *et al*. report that repetitive subconcussive head impacts cause microstructural tissue changes in the developing rat brain, which are detectable with diffusion tensor imaging, and with suggestion of correlates in tissue pathology and behaviour. The model provides a starting point for further characterization of subconcussive head impacts in rodents.

## Graphical Abstract

**Graphical Abstract:**
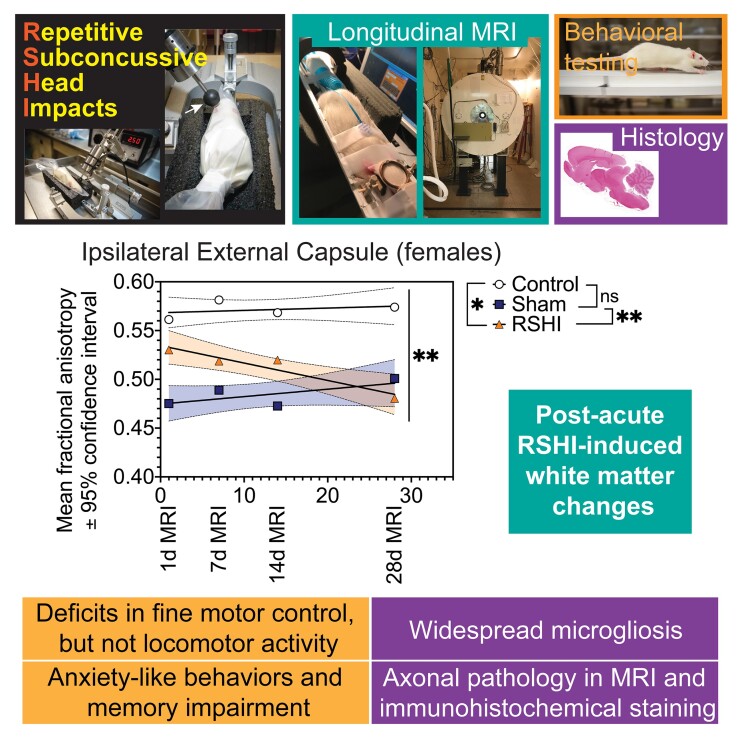

## Introduction

There is growing concern about the potential cumulative effects of sport-related repetitive subconcussive head impacts (RSHIs) on long-term brain health,^1,2^ especially among youth athletes.^3-5^ These repetitive knocks to the head are ‘clinically silent’ as they do not result in diagnosed concussion or overt acute symptoms, but subconcussive impacts still transmit apply force to the brain, which may lead to subclinical pathologic changes. Human studies show that diffusion tensor imaging (DTI) implicates widespread microstructural white matter pathology associated with RSHI,^6,7^ which is in turn associated with worse cognitive performance similar to that seen in patients with traumatic brain injury (TBI).^8-10^ These studies, however, cannot confirm the causal role of RSHI in development of structural and functional effects nor can human studies directly explore tissue injury mechanisms. A reverse translational approach to experimental RSHI in an animal model, however, could characterize pathology of RSHI, identify its imaging correlates and then translate this knowledge to humans by leveraging similar imaging approaches.

Rodent models of TBI are numerous (e.g. see reviews by Shultz *et al*.,^11^ Xiong *et al*.^12^ and Hoogenboom *et al*.^13^) but have only rarely addressed RSHI. Weight drop, which often causes skull fracture and focal injury, and fluid percussion, which requires craniotomy, are inconsistent with the mild degree of injury resulting from sport RSHI. While controlled cortical impact (CCI) entails direct impact on the exposed brain,^14^ a modified CCI procedure (mCCI) with impact to the intact skull or scalp produces neuroimaging findings in rats, which are similar to human mild TBI.^15^ Limitations of the mCCI model include use of anaesthesia and rigid head fixation. Anaesthesia has varied effects that could alter the response to injury^16-18^ and movement of the head following impact is a key feature of the biomechanics of RSHI.^19,20^ Concussion and, to some extent, repeated concussion have been modelled with mCCI, but no studies have reported awake RSHI in animals.

We present results of RSHI in rats, which extends prior work in several ways as follows: (i) animals were fully awake during RSHI; (ii) there was no rigid head fixation for the impact procedure; (iii) the velocity of impact was very low and no overt behavioural abnormalities or gross pathology (e.g. skull fracture or haemorrhage) encountered, which would be typical of more severe injury; (iv) a large number of impacts was administered over multiple days; (v) the time between impacts was short, as is common in sport RSHI; and (vi) adolescent animals were studied, with equal numbers of females and males. Our findings thus advance knowledge beyond concussive injury^13,21,22^ to characterize structural features, functional effects and mechanisms underlying RSHI.

## Materials and methods

All animal procedures were reviewed and approved by the Albert Einstein College of Medicine Institutional Animal Care and Use Committee. Data from 103 adolescent male and female Sprague Dawley rats (Charles River Laboratories, Inc., Wilmington, MA) are included in this report. Groups of two or three same sex animals were housed with *ad libitum* access to food and water on a 12:12 hour light:dark cycle in temperature-controlled animal isolation cabinets (TBJ, Inc., Chambersburg, PA).

### Experimental design

**Figure 1:**
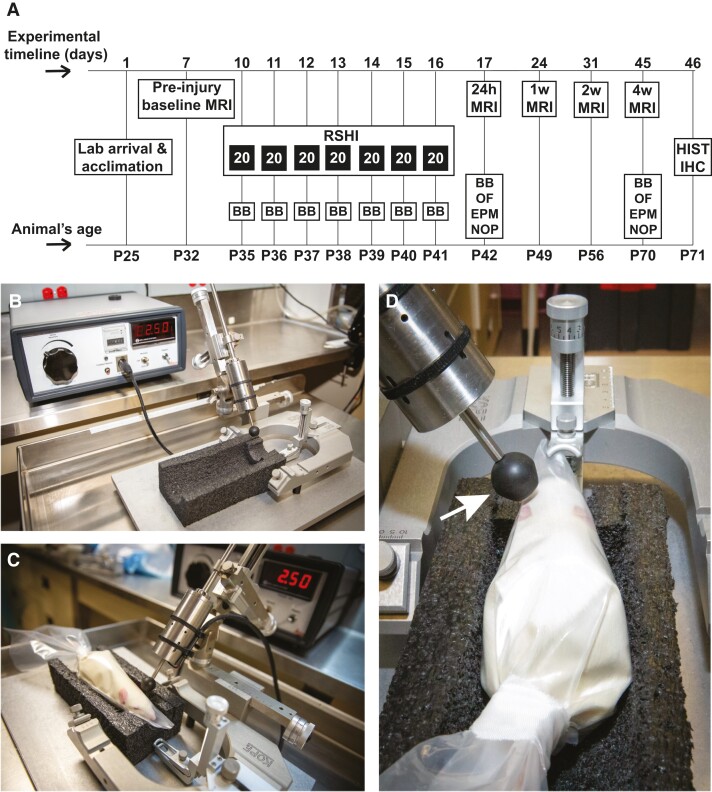
**Study timeline, experimental procedures and apparatus.** (**A**) Study timeline and experimental procedures. After acclimation and baseline MRI acquisition, postnatal Day 35 (P35) animals received 20 subconcussive head impacts per day for 7 consecutive days totalling 140 impacts, with daily BB assessment. One day after completion of the impact protocol, P42 animals underwent 24 hours post-impacts MRI and a larger behavioural assessment, including BB, OF, EPM and NOP. Additional follow-up MRIs were performed at 1, 2 and 4 weeks after final impacts. At 4 weeks post-impacts, animals were behaviourally retested and sacrificed for histology and immunohistochemical analyses. (**B**) Apparatus and experimental setup to generate RSHI in awake animals. (**C**) Awake animals (RSHI and sham) were restrained in a tapered plastic cone, with breathing hole, and subsequently placed in a foam cradle for positioning. No ear bars or other rigid head fixation methods were used to enable head movement after impact. (**D**) A firm rubber-tipped electrostatic impactor was positioned to strike the rat on the intact scalp superficial to the left parietal bone (impact velocity = 2.5 m/s, impact depth = 5 mm, dwell time = 100 ms). Note the white arrow to indicate rubber tip with 10 mm diameter impact surface. RSHI animals received one head impact every 30 s for a total of 20 impacts per day for 7 days, totalling 140 head impacts. Sham-impacted animals received the exact same treatment as trauma animals except the impactor was positioned to remain 1 cm above the animal’s head when fired. Control animals were not restrained or impacted at any time. RSHI, repetitive subconcussive head impacts; BB, beam balance; OF, open field; EPM, elevated plus maze; NOP, novel object placement; HIST, histology; IHC, immunohistochemistry.

The experimental design of this study is summarized in Fig. 1A. Upon arrival in our laboratory at postnatal Day 25 (P25), rats were allowed to acclimate for 1 week. At P32, animals underwent baseline MRI followed by a 3-day recovery before being randomly assigned to one of three groups as follows: (i) RSHI; (ii) sham; or (iii) control. For the next 7 days (P35 to P41), animals underwent daily RSHI (described in more detail in the next section), as well as daily beam balance (BB) assessment. At 24 hours after completion of the impact protocol (P42), animals underwent follow-up MRI and additional behavioural assessment, including BB, open field (OF), elevated plus maze (EPM) and novel object placement (NOP). MRI was repeated at 1 week (P49), 2 weeks (P56) and 4 weeks (P70) post-RSHI. At 4 weeks post-RSHI, animals underwent repeat behavioural assessment (BB, OF, EPM, NOP) and were then euthanized for histology and immunohistochemical analyses. In this work, ‘acute injury phase’ refers to the time between pre-RSHI baseline (P32) until 24 hours post-RSHI (P42), whereas the ‘delayed injury phase’ refers to the time from 24 hours post-RSHI (P42) until 4 weeks post-RSHI (P70).

### Repetitive subconcussive head impact procedure

The experimental setup used to generate RSHI is presented in Fig. 1B. Awake animals were mildly restrained in a flexible, tapered, plastic cone with a breathing hole at its apex (DecapiCones from Braintree Scientific, Inc., Braintree, MA) secured with tape at the open end, which kept the animal in place while allowing movement of the head following impact. The restrained animal was placed in a custom foam cradle with an integrated head rest, but without ear bars, nose cone or other rigid head fixation (Fig. 1C). An electromagnetic impactor (Leica Microsystems, Inc.; Buffalo Grove, IL) fitted with a custom-milled 10 mm diameter, flat impact surface, circular neoprene rubber tip, was positioned to strike the rat on the intact and unshaved scalp over the left parietal bone, off-midline midway between lateral canthus and external auditory meatus (Fig. 1D). Impact parameters, determined in a preliminary dose-escalation study (Supplementary Fig. 1), were velocity = 2.5 m/s, depth = 5 mm and dwell time = 100 ms. Before the start of each impact session, the actuator tip (15° impact angle) was calibrated to ensure the impact location had maximum consistency within and across animals. In the rare occasion that an animal was displaced between impacts (most animals would remain calm and motionless during the impact procedures), we re-calibrated the actuator tip.

RSHI animals received one head impact every 30 s for a total of 20 impacts per day for 7 consecutive days, totalling 140 head impacts. The total time in restraint was no more than 10 minutes per day during the impact protocol. Sham-impacted animals received the exact same treatment as RSHI animals, except that when the impactor was fired, it did not contact the animal. Controls were left undisturbed in their home cages except for weekly handling for cage change. Animals were closely monitored for tolerance of the restraint upon entering the cone, during all impact and sham-impact procedures, and for 30 minutes following release from restraint to assess for changes in behaviour. All animals were weighed prior to restraint on each day of the 7-day RSHI protocol as well as prior to each MRI. In this work, the term ‘post-RSHI’ is used to refer to time beginning after the final impact.

### MRI acquisition and analysis

**Figure 2:**
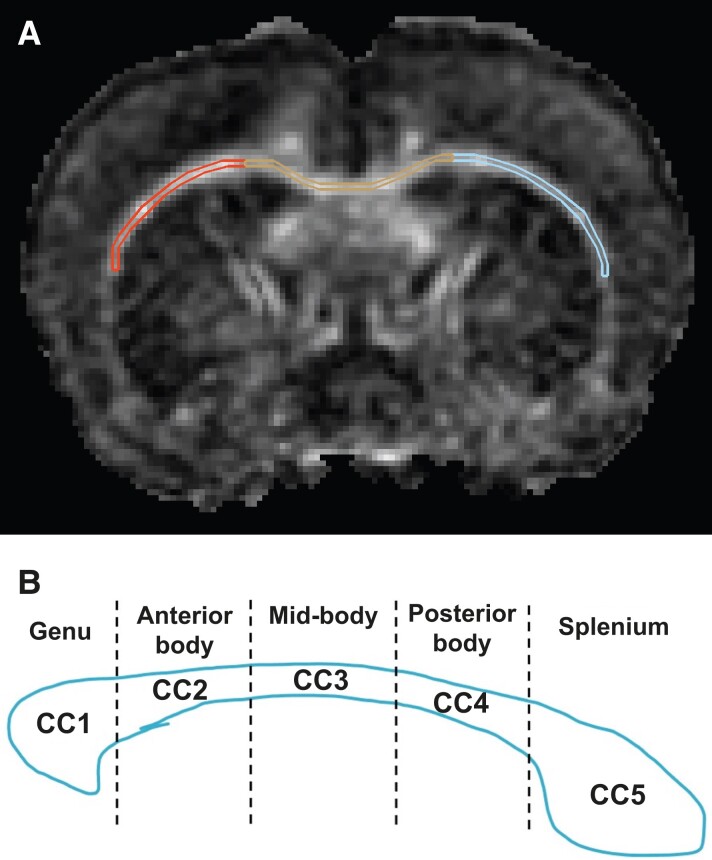
**Corpus callosum and bilateral external capsule regions of interest.** (**A**) Corpus callosum (medial region) and external capsule (bilateral regions) were manually traced in 215 MRI acquisitions on 12 consecutive coronal slices defined by anatomical boundaries adapted from our previously published study^15^ with minor modifications (see Supplementary material for additional details). (**B**) Segmented corpus callosum. To further examine localized changes, each ROI was further divided into five equal sections with the first segment being most anteriorly located and the last segment being most posteriorly located. DTI parameters were extracted as the voxel-weighted average across the regions of interest and used for quantitative analysis. CC, corpus callosum; DTI, diffusion tensor imaging; MRI, magnetic resonance imaging; ROI, region of interest.

A total of 215 brain volumes (DTI and high resolution T_2_-weighted images) were acquired across five time points from 43 animals (8 female/8 male RSHI, 8 female/7 male sham, 6 female/6 male control) at 9.4 T (see Supplementary Figs 2 and 3 for a more detailed description of MRI setup and acquisition parameters used). Animals were endotracheally intubated and mechanically ventilated during MRI with 1.5–1.75% isoflurane in room air (Supplementary Fig. 2). The corpus callosum (CC) and bilateral external capsule (ipsilateral, IEC; contralateral, CEC) were manually traced by three trained raters using MIPAV (v8.0.2) (Fig. 2A). Intraclass correlation coefficients (ICCs) were calculated to validate region of interest (ROI) placement across raters. ROIs were each segmented into five sub-sections along the rostral–caudal axis, for regionally specific analysis (Fig. 2B). The tracing method was adapted from our previously published study^15^ with minor modifications (Supplementary Fig. 3). All tracers were blinded to group allocation. DTI parameters (FA, RD, MD, AD) were extracted as the voxel-weighted average across each ROI.

### Behavioural assessment

A separate cohort of 53 animals (10 female/10 male RSHI, 9 female/10 male sham, 7 female/7 male controls) underwent the exact same impact protocol and was tested at acute and chronic time points (Fig. 1A) for motor coordination and locomotor activity (BB), anxiety, response to novelty, stress, and exploration (OF and EPM) and spatial memory (NOP) (see Supplementary Fig. 4 for a more detailed description of behavioural paradigms used).

### Tissue preparation

Four weeks post-RSHI, 18 animals were randomly selected from the behaviour cohort (3 female/3 male RSHI, 3 female/3 male sham, 3 female/3 male controls) and euthanized for histology and immunocytochemistry analysis. Brains were extracted and immersion-fixed in formalin for one week. Following post-fixation, brains were hemisected along the midline and paraffin embedded. Five micrometre sagittal sections were taken from the ipsilateral (left side) hemisphere, cut from the midline outward on a Leica fully automated rotary microtome (Leica Biosystems, model RM2255, Germany). All histology and immunohistochemistry analyses (described in the next section) were performed in a fully blinded fashion.

### Histology

Sections were stained with haematoxylin and eosin (H&E) and the Bielschowsky silver method. H&E-stained tissue slides were reviewed by an experienced comparative pathologist (Dr. Amanda Beck) for any indication of trauma or other pathology. Thickness of the CC was measured on digitized H&E images at 11 locations 864 µm apart along the rostro-caudal dimension of the CC. Bielschowsky silver stains were reviewed by an experienced veterinary pathologist (Dr. Jerrold M. Ward) for visible neuropathological lesions. See Supplementary Fig. 5 for histological protocols.

### Immunohistochemistry

Immunohistochemical (IHC) stains for ionized calcium binding adaptor molecule 1 (Iba-1), glial fibrillary acidic protein (GFAP), myelin basic protein (MBP), amyloid precursor protein (APP) and paired helical filament-1 (PHF-1) were performed on parasagittal sections, which included corpus callosum, cerebral white matter and cortex (see Supplementary Table 1 for IHC protocols and antibodies used).

### IHC quantification

An automated stain-intensity-based IHC quantification tool was used to analyse the stained sections (CaseViewer, 3DHISTECH Ltd., Budapest, Hungary) (Supplementary Fig. 6). Digitized images were used to segment the CC (CC1 = genu, CC2 = anterior mid-body, CC3 = mid-body, CC4 = posterior mid-body, CC5 = splenium). Additionally, five circular ROIs (1.2 mm^2^) were placed in the cerebral white matter (CTX) halfway between the CC and cortical surface.

### Statistical methods

Stata (Release 13, StataCorp LP, College Station, TX) and GraphPad Prism (v7.03, GraphPad Software, San Diego, CA) were used for statistical analysis and visualization. A random subset of 10 cases was re-traced/re-scored followed by calculation of the ICC for ROI placement and behavioural scoring. All analyses were verified for normality assumptions and parametric or non-parametric tests were used as deemed appropriate. Linear regression was used to obtain rate of change (slope) in DTI metrics for each ROI. Slopes were then compared using one-way ANOVA to assess between-group effects (RSHI versus sham versus control). Linear mixed-effects, paired *t*-test or Wilcoxon matched pair signed rank tests were applied to repeated behavioural measures, and one-way ANOVA or Kruskal–Wallis was used to compare IHC markers. All *post hoc* analyses were corrected for multiple comparisons (Tukey or Dunn as deemed appropriate). All data are presented as mean ± standard error of the mean. Significance was set at *P* < 0.05.

## Results

All rats survived the RSHI procedures. One sham animal did not survive anaesthesia during MRI. During impacts, animals typically remained motionless and did not struggle or vocalize. Following each impact session, rats groomed and ambulated normally. Female sham and RSHI animals gained less weight during the 7-day impact protocol than controls (Supplementary Fig. 7A). All male animals showed similar weight gain, which was greater than for females (Supplementary Fig. 7B and C).

### DTI findings

**Figure 3:**
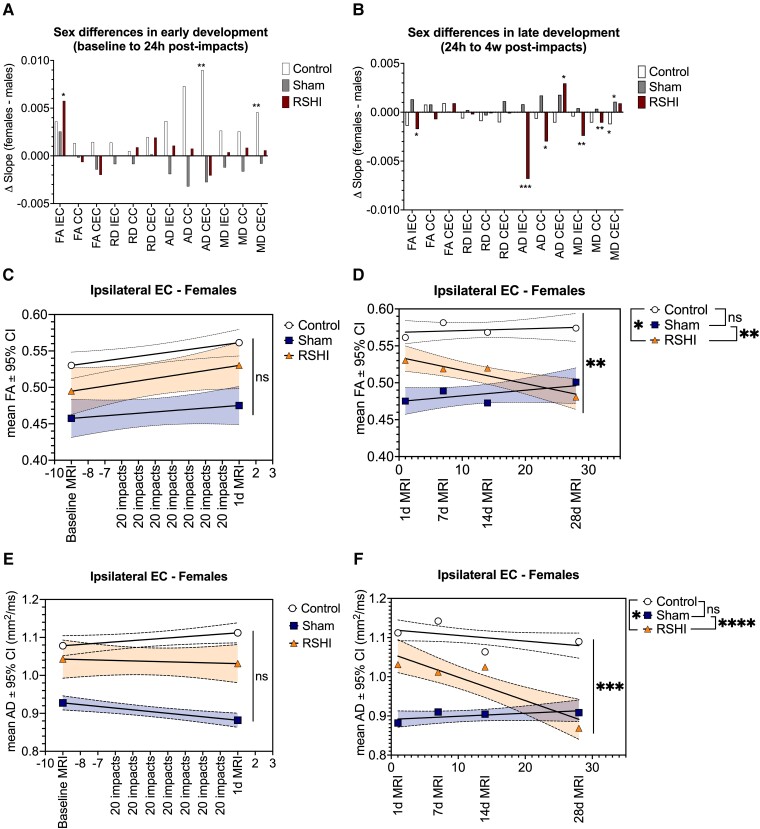
**Sex differences in longitudinal DTI, and long-term FA and AD decline in female RSHI animals.** Sex differences in white matter were detected throughout the study. In the acute injury phase (P32 to P42) (**A**), female controls had, except for RD, exclusively positive linear regression coefficients for all ROIs and DTI metrics, including significant longitudinal FA increases in the IEC (*P* = 0.024), CC (*P* = 0.002) and CEC (*P* = 0.002). In contrast, male controls had non-significant longitudinal FA changes (IEC *P* = 0.837; CC *P* = 0.095; CEC *P* = 0.176), and negative rates of change for MD and AD metrics, which was significantly different from females for the CEC AD (*P* = 0.004) and CEC MD (*P* = 0.008). Both female and male animals had negative RD changes over time. In the RSHI group, we detected a significant difference in IEC with greater FA increase in females compared to males (*P* = 0.032). No sex differences were observed in the sham group. In the delayed injury phase (P42 to P70) (**B**), using simple linear regression, female RSHI animals had greater change in DTI metrics for the IEC FA (*P* = 0.039), IEC AD (*P* < 0.001), CC AD (*P* = 0.042), CEC AD (*P* = 0.033), IEC MD (*P*= 0.002) and CC MD (*P* = 0.005) compared to male counterparts. Given the observed sex differences, DTI trajectories were analysed by sex (**C**–**F**). Among female animals, significant group differences in DTI metrics were primarily observed in the 4 weeks after head impacts (**D**, **F**). Female RSHI animals showed significant FA (**D**) and AD decline (**F**) in the IEC compared to sham and control animals. Control and sham animals exhibited equal trends. See Supplementary Figs 8–17 for additional graphs. Each data point (white = controls, blue/purple = sham, orange = RSHI) in panels **C**–**F** represents the group mean for that MRI assessment. Black straight lines are best fit regression lines with 95% CI. *P < 0.05, **P < 0.01, ***P < 0.001. AD, axial diffusivity; CC, corpus callosum; CEC, contralateral external capsule; CI, confidence interval; DTI, diffusion tensor imaging; EC, external capsule; FA, fractional anisotropy; IEC, ipsilateral external capsule; MD, mean diffusivity; RD, radial diffusivity; ROI, region of interest; RSHI, repetitive subconcussive head impacts.

Intra-rater agreement and inter-rater agreement were excellent for CC [FA ICC = 0.99, 95% confidence interval (CI) = 0.97 to 1.00, *P* = 0.001] and external capsule ROIs (FA ICC = 0.94, 95% CI = 0.71 to 0.99, *P* = 0.001). There were no significant baseline differences in imaging metrics between groups. We identified sex differences in the rate of change of DTI metrics across assessments. In the acute injury phase (baseline to 24 hours post-RSHI), female animals showed greater rate of change for IEC FA (*P* = 0.032), CEC AD (*P* = 0.004) and CEC MD (*P* = 0.008) compared to males (Fig. 3A). In the delayed injury phase (24 hours to 4 weeks post-impacts), sex differences in DTI rates of change were noted for IEC FA (*P* = 0.039), IEC AD (*P* < 0.001), CC AD (*P* = 0.042), CEC AD (*P* = 0.033), IEC MD (*P* = 0.002) and CC MD (*P* = 0.005) (Fig. 3B).

Given the observed sex differences in longitudinal DTI, further group analyses were stratified by sex. Female animals showed group differences in DTI trajectories primarily in the delayed injury phase (24 hours to 4 weeks post-RSHI) for IEC (FA, AD, MD), CC (AD, MD) and CEC (AD, MD, RD) (Fig. 3C–F, Supplementary Figs 8–11). *Post hoc* analysis revealed significant divergence in the slope of longitudinal DTI between RSHI and sham animals for all ROIs tested (*P* < 0.05), except CEC AD (*P* = 0.30) and CEC MD (*P* = 0.76). In contrast to female animals, there were no group differences observed in DTI trajectories among male animals for any ROI, DTI metric or injury phase (Supplementary Figs 12–15).

The preceding analyses examined average measures across relatively large ROIs (whole CC and EC). To identify localized changes, we separately examined rostral–caudal sub-segments of the CC and EC (Supplementary Figs 16 and 17). Of 75 sub-segments assessed, the anterior mid-body of the CC (CC2) and mid-EC (IEC2, CEC2), which are located in the same coronal plane, was most often affected (10 out of 40 segments, 25%). Three comparisons (bilateral EC AD and IEC MD) showed group differences at all five sub-segments.

### Behavioural assessments

Excellent intra-rater reliability was established for foot fault scoring on the BB (ICC = 0.94, 95% CI = 0.75 to 0.98, *P* < 0.001) as well as for scoring time in the NOP test (ICC = 0.92, 95% CI = 0.71 to 0.98, *P* < 0.001). Reliability testing was not performed for the OF and EPM as these tests were analysed using video tracking software.

#### BB

**Figure 4:**
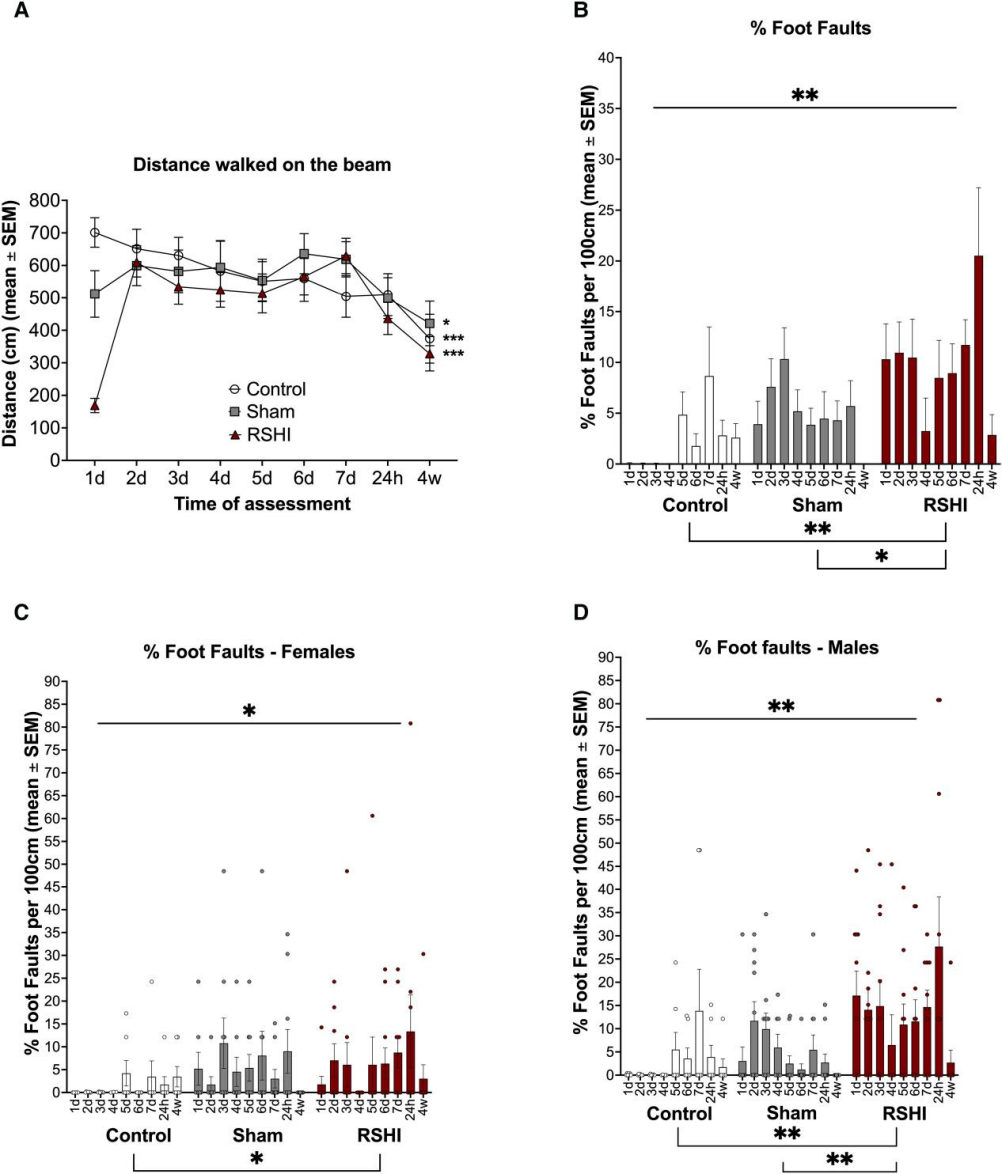
**Beam walking assessments at acute and chronic time points following RSHI.** (**A**–**D**) Beam walking was assessed daily during the impact protocol and at 24 hours and 4 weeks after the final day of head impacts. Mixed-effects analysis showed that all animal groups travelled less over time, but there were no between-group differences in mean distance travelled (**A**). Each data point in Fig. 4A represents the group mean for that respective time assessment. Following correction for distance (i.e. percentage foot faults per 100 cm beam distance), nested one-way ANOVA showed that there were significant group differences in percentage of foot faults committed on the beam (**B**). *Post hoc* Tukey’s revealed that RSHI animals exhibited significantly more foot faults than sham and control animals. After stratification by sex (**C**, **D**), female RSHI made more foot faults than female controls, but not female sham (**C**). Male RSHI made more foot faults than male controls and male sham (**D**). Male RSHI exhibited more foot faults than female RSHI. Each data point in panels **C** and **D** represents the value of an individual animal.

There were significant within-group differences in distance travelled on the beam over time (RSHI *P* < 0.0001, sham *P* = 0.034, control *P* = 0.004). Mixed-effects analysis with *post hoc* Tukey’s showed that controls travelled less at 4 weeks versus Day 1 (*P* = 0.004), RSHI travelled less at 4 weeks versus Day 2 (adj. *P* < 0.0001), but not Day 1. Sham travelled least at 4 weeks, but this was not significantly different from other time points. There were no between-group differences in total distance travelled (Fig. 4A). The percentage foot faults per 100 cm travelled was significantly different between groups (*P* = 0.002) (Fig. 4B). *Post hoc* Tukey’s revealed that RSHI animals made more foot faults (9.9%-foot faults/100 cm) than sham (5.0%-foot faults/100 cm; *P* = 0.033) and controls (2.4%-foot faults/100 cm; *P* = 0.002). There were no differences in foot faults between control and sham animals (*P* = 0.37). Furthermore, a greater proportion of RSHI animals (12/20 = 60%) made foot faults on the final day (Day 7) of the RSHI protocol than sham (5/19 = 26%) and controls (3/13 = 23%) (*P* = 0.04).

After stratification by sex, group differences remained significant (females *P* = 0.046, males *P* = 0.0009, Fig. 4C and D). Female RSHI made more foot faults than female controls (*P* = 0.047), but not female sham (*P* = 0.92) (Fig. 4C), whereas male RSHI made more foot faults than male controls (*P* = 0.002) and male sham (*P* = 0.004) (Fig. 4D). Additionally, male RSHI (13.7%-foot faults/100 cm) made more than twice as many foot faults as female RSHI (6.1%-foot faults/100 cm) (*P* = 0.01) (Fig. 4C and D).

For all groups, most foot faults (52–65%) occurred on the narrowest section of the beam (interval 3) (RSHI *P* = 0.0009; sham *P* = 0.001; controls *P* = 0.08), which was not significantly different when compared between groups or sex (*P* > 0.05) (Supplementary Fig. 1^8^).

#### OF

**Figure 5:**
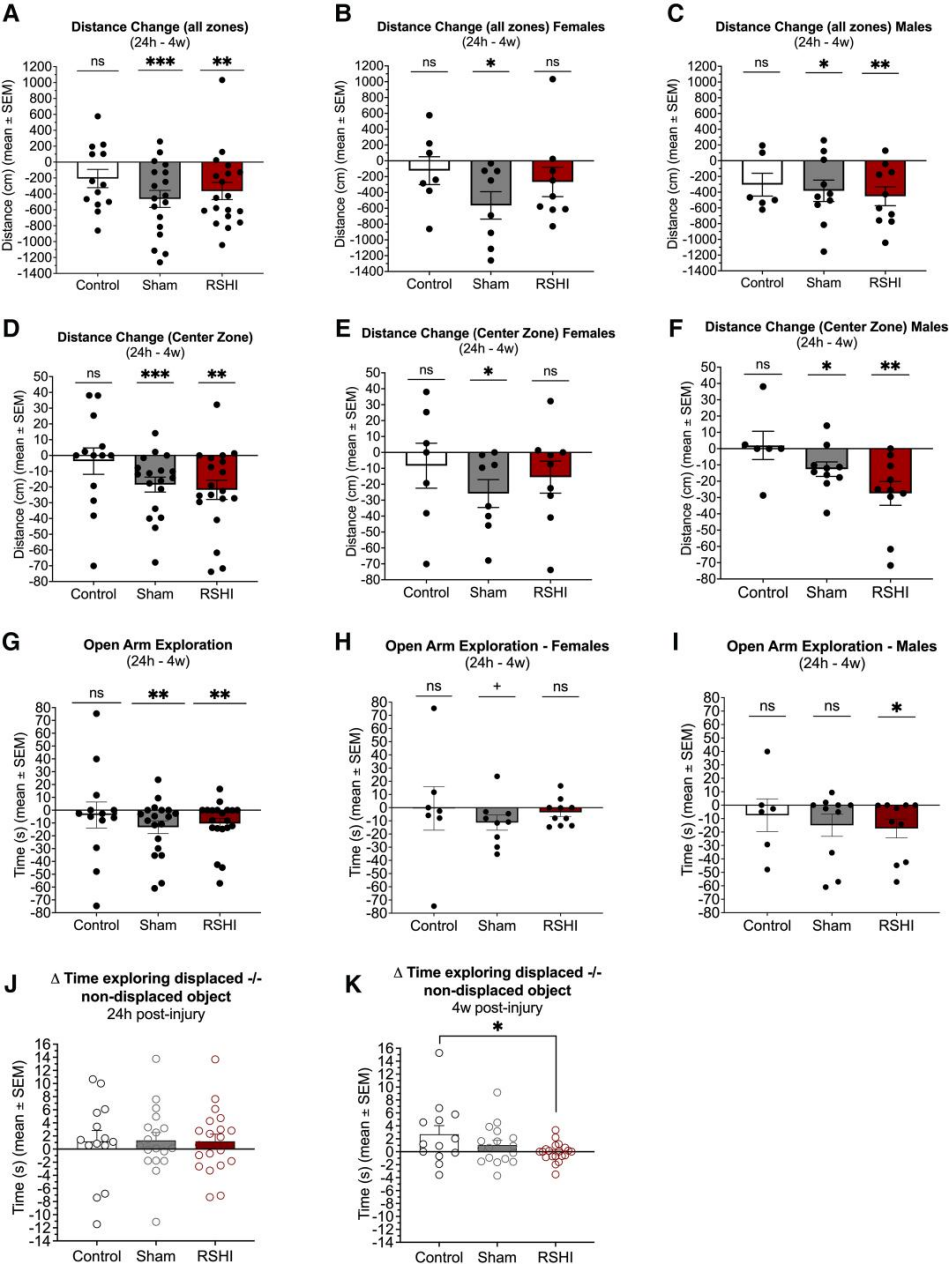
**Open field, elevated plus maze and novel object recognition assessments at acute and chronic time points following RSHI.** (**A**–**F**) Open field test at 24 hours and 4 weeks post-impacts. Repeated measures revealed that locomotor activity (all zones) decreased significantly over time for RSHI animals (−42.5%) and sham (−52.6%), but not controls (−27.8%) (**A**). After stratification by sex (**B**, **C**), this effect remained significant for male RSHI, male sham and female sham, but not female RSHI. Similarly, centre zone locomotor activity decreased significantly over time for RSHI (−75.5%) and sham (−69.4%), but not controls (−14.9%) (**D**). After stratification by sex (**E**, **F**), this effect remained significant for male RSHI, male sham and female sham, but not female RSHI. Each data point in panels **A**–**F** represents the value of an individual animal. (**G**–**I**) Elevated plus maze assessment at 24 hours and 4 weeks post-impacts. Repeated measures revealed that relative to controls, RSHI and sham animals spent significantly less time in the open arms at 4 weeks compared to 24 hours post-impacts (−65.5% and −81.3%, respectively) (**G**). After stratification by sex, significant decrease in open arm exploration was observed in male RSHI (**H**) but not female RSHI (**I**). Each data point in panels **G**–**I** represents the value of an individual animal. (**J**, **K**) Novel object placement test at 24 hours and 4 weeks post-impacts. At 24 hours, there were no group differences in displaced object versus non-displaced object exploration (**J**). However, at 4 weeks, RSHI animals spent less time exploring the displaced object than control animals (one-way ANOVA with Tukey’s multiple comparison’s test) (**K**). Each data point in panels **J** and **K** represents the value of an individual animal. ^+^*P* < 0.10, *P < 0.05, **P < 0.01, ***P < 0.001. SEM, standard error of the mean.

Locomotor activity (all zones) declined from 24 hours to 4 weeks for RSHI (−42.5%, *P* = 0.003) and sham (−52.6%, *P* = 0.0005), but not for controls (−27.8%, *P* = 0.10) (Fig. 5A). After stratification by sex (Fig. 5B and C), this effect remained significant for male RSHI (*P* = 0.004), male sham (*P* = 0.027) and female sham (*P* = 0.014), but not female RSHI (*P* = 0.19). Similarly, centre zone locomotor activity decreased for RSHI (−75.5%, *P* = 0.002) and sham (−69.4%, *P* = 0.0007) but not controls (−14.9%, *P* = 0.82) (Fig. 5D). After stratification by sex (Fig. 5E and F), this effect remained significant for male RSHI (*P* = 0.004), male sham (*P* = 0.027) and female sham (*P* = 0.021), but not female RSHI (*P* = 0.15).

#### EPM

Time spent in the open arms declined from 24 hours to 4 weeks for RSHI (−65.5%, *P* = 0.010) and sham (−81.3%, *P* = 0.009), but not controls (−19.1%, *P* = 0.52) (Fig. 5G). After stratification by sex (Fig. 5H and I), this effect remained significant for male RSHI only (*P* = 0.016). General locomotor activity (total distance travelled) was not different between groups for any time (*P* > 0.05).

#### NOP

At 24 hours post-RSHI, there were no group differences in displaced versus non-displaced object exploration (Fig. 5J). At 4 weeks, controls and sham spent more time exploring the displaced object (+134.3% and +42.8%, respectively), while RSHI animals spent 3.6% less time exploring (*P* = 0.047) (Fig. 5K).

### Histology

No abnormalities, including evidence of haemorrhage or contusion, were observed on H&E sections (Supplementary Fig. 19). No visible lesions were detected on Bielschowsky silver stain, and there were no clear sex-related differences in histological or IHC data.

#### Thickness of the corpus callosum

**Figure 6:**
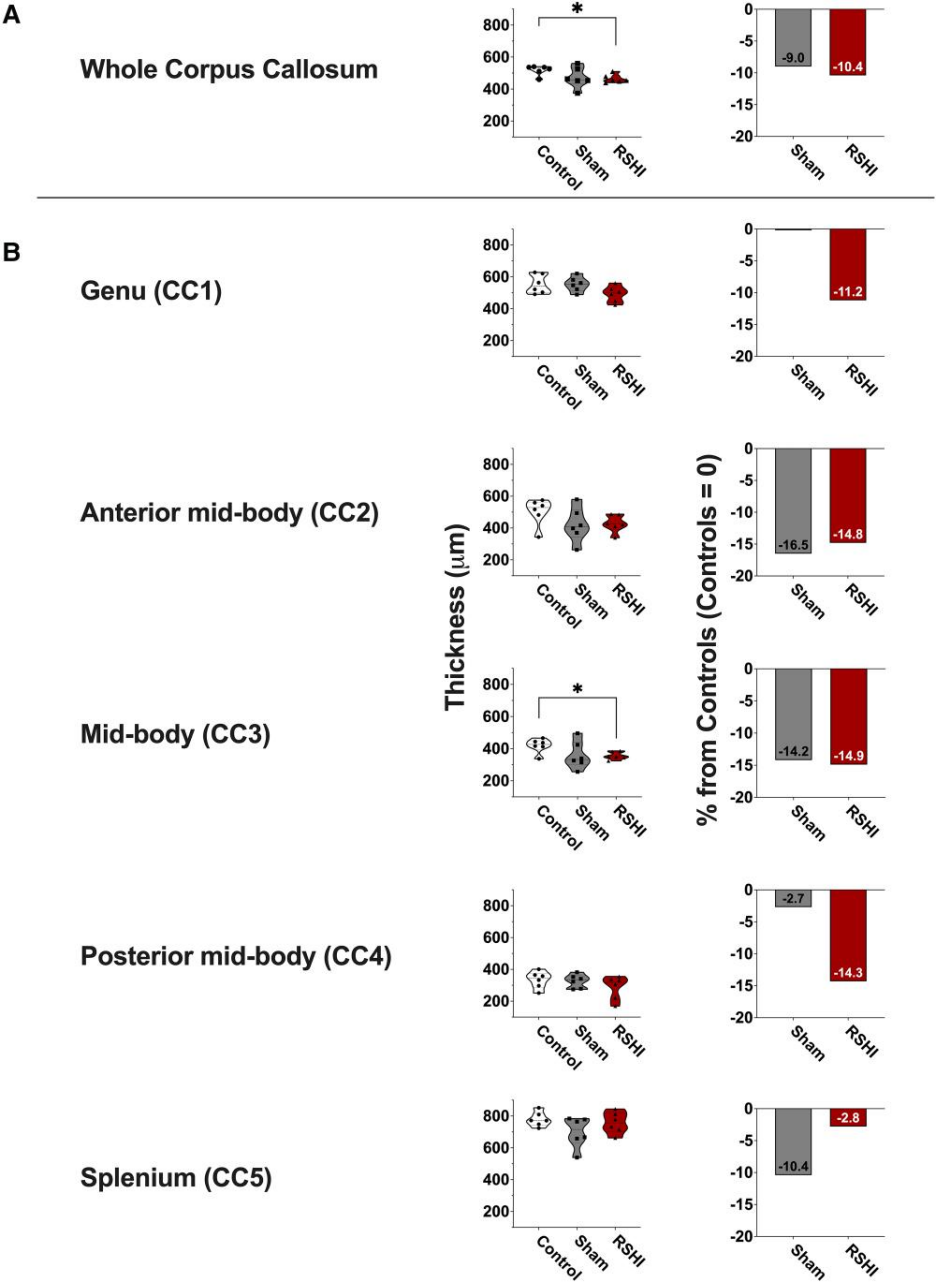
**Corpus callosum thickness.** The average thickness of the whole corpus callosum was smaller in RSHI animals (−10.4%) and sham animals (−9.0%) relative to controls, statistically significant for RSHI animals only (Kruskal–Wallis test with Dunn’s *post hoc*) (**A**). After segmentation, RSHI animals had 2.8% to 14.9% thinner corpus callosum compared to controls, statistically significant for CC3 (Kruskal–Wallis test with Dunn’s *post hoc*) (**B**). Left column = absolute thickness (micrometres), right column = relative difference from controls (control = 0%). *P < 0.05. CC, corpus callosum. Each data point in panels **A** and **B** represents the value of an individual animal.

Compared to controls, CC measured on H&E sections was thinner in RSHI and sham animals (−10.4% and −9.0%, respectively), but reached significance only in RSHI (*P* = 0.026) (Fig. 6A). RSHI CC thinness, compared to controls, varied (−2.8% to −14.9%) along the rostral–caudal dimension. The difference was greatest in the mid-body at CC3 (*P* = 0.038) (Fig. 6B).

### Immunohistochemistry

#### Iba-1

**Figure 7:**
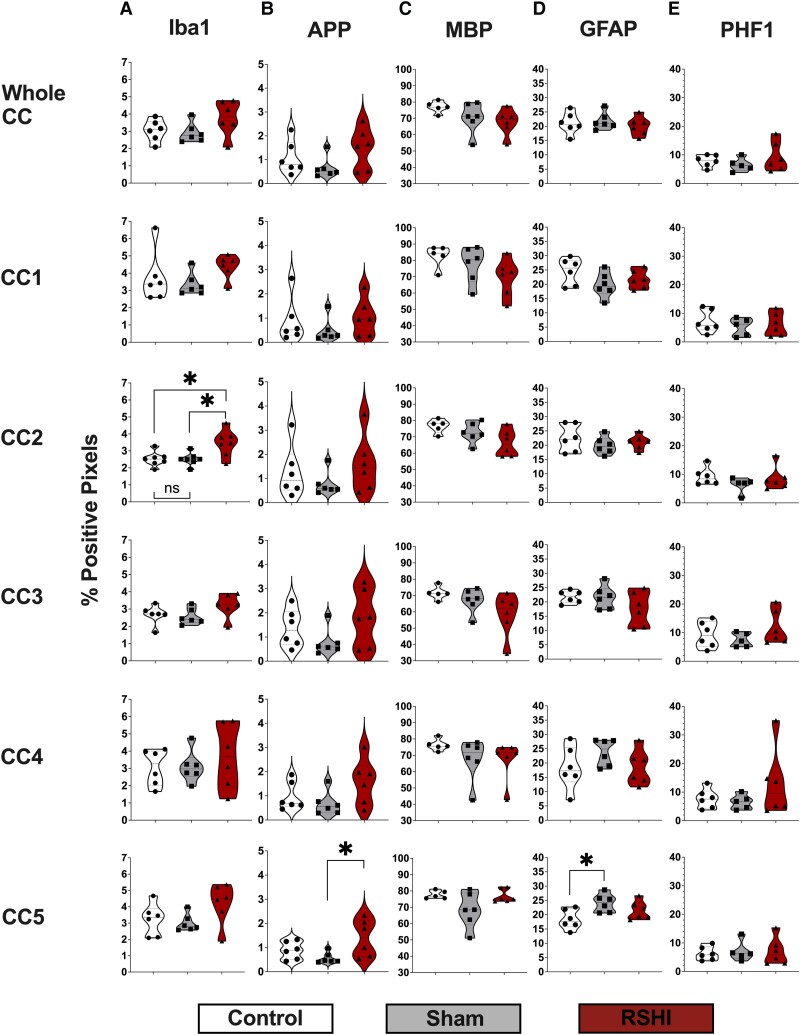
**Immunostaining of the corpus callosum.** RSHI animals had higher levels of Iba-1 staining in all regions of the corpus callosum compared to sham and control animals (+17% to +38% higher than control levels) (**A**), statistically significant for CC2. One-way ANOVA with *post hoc* Tukey’s revealed increased Iba-1 staining for RSHI versus control and RSHI versus sham, but not control versus sham. RSHI animals had higher levels of APP staining in all CC regions than the other groups (up to 65% higher than controls) (**B**), statistically significant for CC5 (*P* = 0.038). One-way ANOVA with *post hoc* Tukey’s revealed significantly higher APP immunostaining for RSHI versus sham (*P* = 0.039), but not RSHI versus control (*P* = 0.83). RSHI animals had lower levels of MBP staining in all corpus callosum regions compared to control animals (−1% to −18% lower than control levels), although this did not reach statistical significance (**C**). No between-group differences in GFAP (**D**) or PHF-1 (**E**) were noted. *P < 0.05. CC, corpus callosum; Iba1, ionized calcium binding adaptor molecule 1; APP, amyloid precursor protein; MBP, myelin basic protein; GFAP, glial fibrillary acidic protein; PHF-1, paired helical filament-1. Each data point in panels **A**–**E** represents the value of an individual animal.

**Figure 8:**
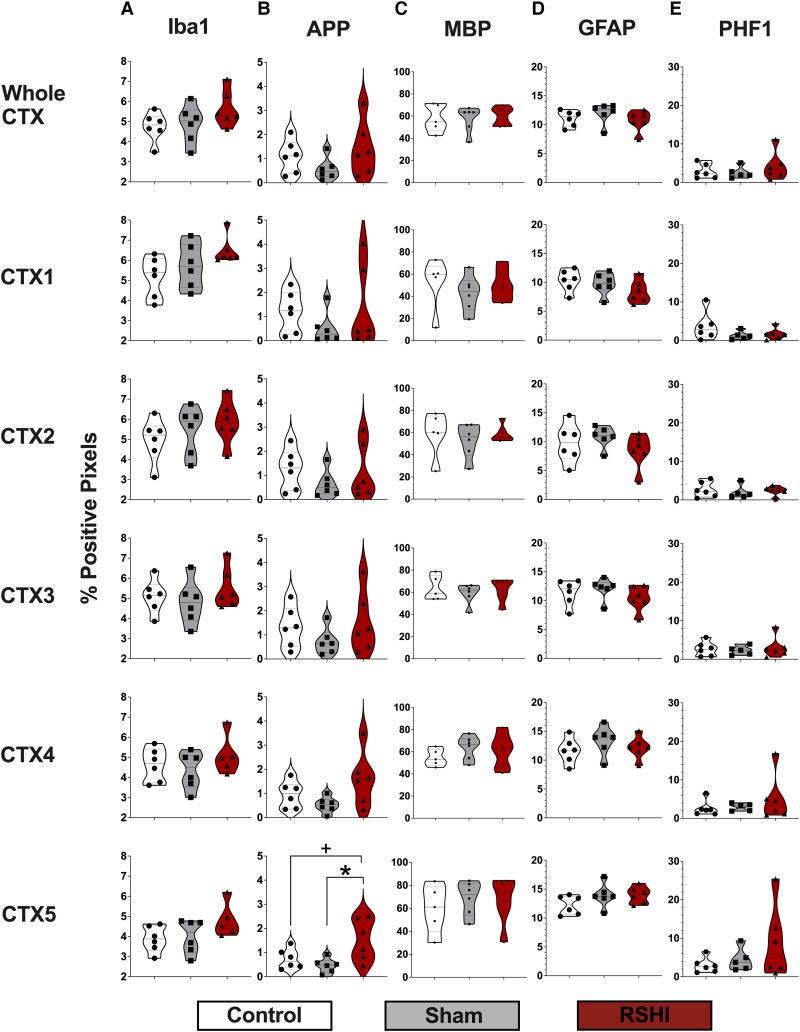
**Immunostaining of cortical white matter regions.** RSHI animals had higher levels of Iba-1 staining in all sampled regions of the cortical white matter (+8% to +25% higher than control levels) (**A**), although this did not reach statistical significance. There were no differences in Iba-1 staining between control and sham animals for any region. RSHI animals had higher levels of APP (up to 110% higher than controls) in all sampled CTX regions, except CTX2, which reached significance for CTX5 versus sham (*P* = 0.016), and borderline significant versus controls (*P* = 0.056) following one-way ANOVA with Tukey’s multiple comparisons test (**B**). There were no differences between sham and controls for any region. No between-group differences in MBP (**C**), GFAP (**D**) or PHF-1 (**E**) were noted for the cortical white matter regions. *P < 0.05, ^+^*P* < 0.10. CTX, cerebral white matter. Each data point in panels **A**–**E** represents the value of an individual animal.

RSHI animals exhibited trends towards greater Iba-immunostaining in all CC regions (Fig. 7A), which reached significance for the anterior-body CC2 (*P* = 0.006). *Post hoc* Tukey’s revealed more Iba-1 staining for RSHI versus control (*P* = 0.013) and RSHI versus sham (*P* = 0.012). Similarly, RSHI animals had higher levels of Iba-1 in all sampled CTX regions compared to sham and control (up to 25% higher than controls), but this did not reach statistical significance (*P* > 0.08) (Fig. 8A). No differences between sham and control were observed for any region.

#### APP

RSHI animals exhibited trends towards greater APP immunostaining in all CC regions compared to sham and control (Fig. 7B), which reached significance for the splenium CC5 (*P* = 0.038). *Post hoc* Tukey’s revealed significantly higher APP for RSHI versus sham (*P* = 0.039), but not RSHI versus control (*P* = 0.83). Similarly, RSHI animals exhibited greater APP immunostaining (up to 110% higher than controls) in multiple CTX regions versus sham (e.g. CTX5; *P* = 0.016), but not versus controls (CTX5, *P* = 0.056) (Fig. 8B). We found no differences between sham and controls for any region (*P* > 0.05).

#### MBP

RSHI animals exhibited trends towards less MBP immunostaining in several CC regions (i.e. anterior-body CC2, *P* = 0.054; mid-body CC3, *P* = 0.099; and posterior-body CC4, *P* = 0.096); compared to controls (up to 18% lower), but not sham (Fig. 7C). No differences were detected in CTX (*P* > 0.05) (Fig. 8C). Sham and controls did not differ for any region (*P* > 0.05).

#### GFAP

There were group differences in GFAP immunostaining at the splenium CC5 (*P* = 0.029) (Fig. 7D). *Post hoc* testing indicated higher levels of GFAP staining for sham versus control (*P* = 0.023), but not sham versus RSHI (*P* = 0.40) or RSHI versus control (*P* = 0.25). No differences were detected in CTX (*P* > 0.05) (Fig. 8D).

#### PHF-1

There were no group differences in PHF-1 staining for CC (Fig. 7E) and CTX regions (Fig. 8E) (*P* > 0.05).

## Discussion

In this study, we performed highly repetitive RSHI, with 140 impacts to the head over a 1-week period, far more than in any previously reported animal model of repetitive TBI to date.^13^ Importantly, the animals were not anaesthetized and tolerated the impact protocol with no overt changes in behaviour. No overt stigmata of TBI, such as haemorrhage or skull fracture, were caused by the repeated impacts. We detected white matter changes using DTI among female adolescent rats, whose developmental white matter trajectories diverged from sham and control animals during follow-up. We also found behavioural changes, primarily acute transient motor control deficits, in RSHI, but not sham or control. Notwithstanding absent signs of gross injury, we observed trends in the data for CC thinning and neuropathological changes detected by quantitative IHC, which reached significance for some regions. Our findings suggest that RSHI causes sex-specific microstructural tissue changes detectable by DTI, which are associated with histopathological effects, as well as minor behavioural differences between sham and RSHI.

### Primary features of our experimental approach

While most studies have used anaesthetics during trauma induction,^13^ we studied awake animals. This approach was intended to mitigate confounding by anaesthetic effects,^16-18^ particularly given our multi-day head impact protocol. The use of awake animals has been reported before^23-26^ and should be considered in future studies. Elimination of anaesthetics introduces stress due to repeated restraint and injury, which is a potential confound. Repeated stress during or followed by mild TBI has been associated with altered brain metabolism and more severe behavioural deficits in rats compared to those that experience mild TBI alone.^27^ However, stress is also a cofactor in human RSHI, certainly more so than repeated general anaesthesia.

The magnitude and number of head impacts and short inter-impact interval we applied differ from almost all studies of mild head trauma in rodents. We chose parameters in light of human contexts of RSHI, such as sport. More severe impacts with lower frequency, such as a 24 hour inter-impact interval, may be appropriate for modelling repetitive concussion, but are less consistent with human RSHI, where impacts are typically spaced much more closely.^6,8,9,28,29^

### Acute RSHI-induced alterations of white matter development

Linear DTI trajectories in RSHI animals significantly diverged from control animals, but only among females. Female RSHI animals exhibited decline of AD and MD compared to increase among female controls. Both AD and MD DTI metrics have been widely used to assess axonal degeneration in the CNS.^30^ AD is thought to reflect axonal integrity and may therefore provide insight into underlying pathology of white matter injury.^31,32^ Female control animals exhibited AD increase, greater restriction of diffusion across the fibre, consistent with axonal growth and development. This effect was lacking in female RSHI animals, which showed AD decline, consistent with axonal injury. Both male and female RSHI animals showed significant longitudinal RD decreases consistent with myelin development, similar to controls and shams.

MD, representing the direction-independent magnitude of diffusion, is an overall measure of white matter microstructural integrity. Observed MD decreases in RSHI animals were likely the result of a complicated interplay between developmental changes (i.e. RD decreases) and axonal injury (i.e. AD decreases). During the acute injury phase, cell swelling would manifest as lower MD due to compression of the extracellular space. However, additional histopathological characterization is required to better understand the neurobiological correlates of our DTI measures.

Notably, DTI trajectories in RSHI and sham did not differ, suggesting that effects of stress could account for most of the group differences observed in the acute injury phase. As described below, trajectories of RSHI and sham do diverge at later time points.

### Post-acute RSHI-induced alterations in white matter development

While some DTI trajectories diverged acutely (above), most differences, significant only in female animals, were seen in the weeks following final impact. The reason for sex-divergent responses to RSHI is unclear, but in line with recent findings that females exhibit more widespread evidence of microstructural white matter alterations related to RSHI than males.^7^ Our findings add to the growing body of literature on sex differences in response to brain injury, as reported in both human^33-37^ and animal studies^38^ of TBI.

In contrast to control and sham, female RSHI animals exhibited a strong linear decrease in external capsule FA ipsilateral to the impact site (IEC), consistent with animal studies of repetitive mild traumatic brain injury (mTBI)^39,40^ and with studies of soccer heading.^6^ Low FA is consistent with microstructural pathology due to RSHI, although an interaction between stress and RSHI should be a focus of further investigation.

RSHI animals exhibited decline of AD and MD over time, which was not seen in control or sham animals. Moreover, greater APP immunostaining in female RSHI compared to sham and control suggests that RSHI causes axonal pathology that is not accounted for by stress alone. Axonal injury following RHI has been shown in animal models of more severe TBI,^41,42^ but not after RSHI.

While RSHI animals showed age-dependent decrease in RD, consistent with development and similar to the other groups, subtle changes in myelin development cannot be excluded. In a separate analysis, we found that the magnitude of RD change 1 month after RSHI (12% lower than baseline) did not reach control (20% lower than baseline) or sham (18% lower than baseline) levels (Supplementary Fig. 20). While preliminary, this subtle change in RD is consistent with a trend towards lower MBP immunostaining in RSHI animals, though this finding did not reach significance in our small IHC sample. The possibility that RSHI induces both axonal pathology and delay of myelination or causes myelin injury is thus an important focus for future investigation.

Our findings across ROIs support a diffuse but non-uniform pattern of injury. DTI changes were detectable bilaterally, but greatest ipsilateral to the impact. In addition, effects declined within each region (EC, CC) along the rostral–caudal access from the impact site. These findings may reflect propagation and dissipation of forces from the point of head impact.

### Stress induced alterations in white matter development

The effect of stress was not a primary focus of this study. However, we encountered DTI changes in sham animals that diverged from controls. Because sham animals experienced restraint-related stress only and no physical trauma, we believe that these effects reflect stress alone. Our restraint method has long been used in rodent studies as a means of producing and studying the effects of stress.^43^ To minimize the confounding effects of stress, we limited the amount of time the animals were exposed to restraint stress and used an additional set of controls to account for possible changes as a result of stress alone. Few preclinical DTI studies have reported measurable effects of stress.^44-47^ These studies, however, employed extended exposure (hours) to multiple stressors for extensive periods of time (8 weeks or longer), often examining male adult rats only. While a secondary end-point, our findings suggest that short bouts of restraint-related stress may be sufficient to induce microstructural alterations detectable by DTI. Further study, considering additional measures, such as cortisol, can further clarify the role of stress on brain microstructure in the context of RSHI.

### RSHI induces acute and transient deficits of fine motor control, but not of locomotor activity

Despite normal locomotor activity, RSHI animals exhibited subtle deficits in fine motor function compatible with a subconcussive level of injury. This correlates, for example, with acute mild motor deficits described in soccer RSHI^10,48^ and sparring bouts in boxers, in the absence of recognized mTBI.^49^ Foot faults were mostly observed during the 7-day impact protocol and returned to sham/control levels 4 weeks after RSHI, suggesting an acute and transient phenomenon. Since we did not measure motor function between 24 hours and 4 weeks post-RSHI, it may be that motor function recovered even more rapidly, as has been reported in repeated TBI.^50-56^

### Both RSHI and sham induce persistent anxiety-like behaviours

Both RSHI and sham groups, but not controls, exhibited significantly less exploration of the OF centre zone and the EPM open arms at 4 weeks compared to 24 hours post-RSHI, consistent with persistent anxiety-like behaviour as reported in repeated mild TBI.^23,57-59^ Although our study was not conceived to explicitly address stress as an outcome, these findings in sham animals suggest neuropathological sequalae of repetitive stress.

### Short-term memory performance

RSHI animals spent less time exploring the displaced object in the NOP test at 4 weeks post-impacts than control animals, but these effects were not different from those seen in the sham condition. While memory impairment has been reported in adult and juvenile rodent models of repeated TBI,^54,60-65^ larger study samples are needed to demonstrate more clearly if memory-related injury effects occur as a result of RSHI.

### Sex differences on behavioural assessments

Many rodent TBI studies have underrepresented females.^13,66^ However, with growing evidence for sex-dependent differences in risk and susceptibility to RSHI from human^7^ and animal studies,^38^ it is imperative that studies include both sexes. In contrast to the predominance of DTI effects in female RSHI, we found that male rats in general performed worse than female rats on most behavioural assessments. First, female rats typically ambulated more than males, especially in the OF, a common finding in studies employing this paradigm.^67,68^ Second, on the BB, males performed worse than females, regardless of group. These findings are in accordance with prior studies of experimental TBI demonstrating better performance by female rats on motor tasks, including BB and the Morris water maze,^38,69,70^ although some studies report no sex differences or better performance for males.^66^ Third, anxiety-like behaviours were apparent in RSHI and sham animals, with the effects in RSHI animals mostly driven by males. This finding contrasts with findings in humans, where females suffer more severe and protracted TBI- and RSHI-related anxiety and depression than do males.^33,66^ Results of prior experimental studies are consistent with our findings, reporting a protective effect of female sex on depression- and anxiety-related behaviours following TBI.^38^ Other studies report contrasting findings following weight drop with rotation.^71^ The paucity and diversity of studies to date preclude any definitive conclusions.

### Thinning of the corpus callosum in RSHI and sham-injured animals

Prior reports have indicated CC thinning after more severe experimental TBI.^72-74^ While we found the CC thickness in both RSHI and sham rats to be ∼90% of that of the control rats at 4 weeks post-impacts, there were no significant differences between RSHI and sham animals. For this reason, thinning of the corpus callosum cannot be determined as a sole effect of head injury in our study. Further research with larger study samples is needed to separate RSHI from stress-related effects.

### Immunohistochemical findings following RSHI

While microgliosis has been widely reported in rodent studies of repeated mild TBI,^41,42,53,55-57,75-79^ we are not aware of similar published findings in RSHI, except one study^80^ that employed a lateral fluid percussion injury, which may not be consistent with the mild degree of injury resulting from closed-head impacts seen in sports. We did not find evidence for astrogliosis, which is in line with a report of RSHI in tau transgenic mice.^81^ It is possible that RSHI may have a unique neuropathological signature primarily characterized by microgliosis, not astrogliosis. Alternatively, the timing of assessment (i.e. 4 weeks post-impacts) may have been outside the window of detectable GFAP changes.^82^

We generally found trends in the data for myelin changes in RSHI in line with prior reports of myelin changes in adult rodents within one month following mild to severe TBI.^83-85^ Our findings are exploratory in nature and do not reach statistical significance, but may guide future RSHI studies using IHC. RSHI-induced microgliosis may be responsible for myelin changes.^74^ TBI-induced microgliosis results in activation and polarization of the M1 microglial phenotype, with release of high levels of proinflammatory cytokines that in turn hinder axonal regeneration and oligodendrocyte maturation.^79,86,87^ As mature oligodendrocytes myelinate axons in the CNS, increased microglial activation may thus be responsible for myelin changes. We also noted a spatial gradient of MBP change along the length of the CC with the greatest magnitude of effect in the mid-body of the CC, congruent with the location of head impact. Diminished MBP remote from the impact location suggests a diffuse injury and is consistent with our DTI findings of attenuated developmental trajectories of RD in RSHI animals.

We observed consistent trends for excess APP staining in RSHI, which reached significance for the posterior segments of the corpus callosum and cortical white matter, consistent with axonal pathology and in line with two prior repeat mild TBI studies.^52,75^ The presence of APP 1 month following RSHI indicates persistent neuropathology long after the head impacts have ceased. This is the first study to report such changes following RSHI. MRI findings of widespread AD changes following RSHI (above) provide a correlate to these APP results.

Because repetitive TBI has been linked to the development of chronic traumatic encephalopathy (CTE),^88,89^ we performed immunostaining for hyper-phosphorylated tau, which is observed in post-mortem brain specimens of CTE patients.^90^ We did not find evidence for tau deposition related to RSHI.

## Limitations

Our findings should be considered in light of several limitations. First, due to the awake state of animals and non-rigid head fixation used in our model, some variation in head impact location across animals cannot be excluded. We believe that this variation was mitigated by careful positioning of animals, repeated calibration of actuator tip in addition to the relatively large surface of the rubber impact tip used. Moreover, some variability in impact location would in any case be consistent with real-life repetitive head impacts. Second, while our impact parameters were carefully chosen based on a preliminary dose-escalation study, additional work to explore and optimize impact parameters (e.g. impact depth and impact frequency) can further define the model that is most consistent with RSHI. Third, although DTI is sensitive to white matter changes, it is not specific to a particular pathological mechanism. It is unclear, for example, if changes in FA are the result of abnormalities of myelin, axolemma, inflammation or other factors.^91^ Therefore, our suggestions regarding mechanistic specificity of the imaging measures should be considered preliminary, but can inform hypotheses and guide further histopathological characterization. Fourth, our sample for histopathological/IHC analyses was relatively small, and stains were limited to mid-sagittal tissue sections. Despite small sample size, we demonstrated strong trend data that support further study in larger numbers and at additional regions of interest. Fifth, our study focused on RSHI-induced brain and behavioural changes during adolescence, but future RSHI studies are needed to assess these changes into adulthood. Finally, we used rats for our model, which has clear economical, practical and ethical benefits, but also presents translational challenges to human brain injury due to differences in brain size, anatomic features such as the falx cerebri and tentorium cerebelli and head impact biomechanics. Although the biomechanical equivalencies between rodent models of RSHI and human RSHI are limited, our model provides a starting point for further characterization of RSHI effects in rodents and, potentially, future investigations in larger gyrencephalic animals.

Despite inherent limitations and translational challenges associated with simulating human RSHI in rats, the significant contribution of this work is to demonstrate in awake animals that a very mild repetitive hit—much lower in magnitude, but much more frequent than what has been used to induce concussion—causes structural and functional changes that warrant further investigation.

## Conclusions

RSHI may cause persistent, functionally significant, microstructural tissue changes in the developing rat brain, which are detectable with DTI and with suggestion of correlates in tissue pathology and behaviour. The congruence of our imaging findings with reports of human RSHI suggests that neuroimaging can serve as a translational bridge to advance study of injury mechanisms and development of interventions.

## Supplementary Material

fcad316_Supplementary_Data

## Data Availability

The data that support the findings of this study are available from the corresponding author, upon reasonable request.
