# Supplementary material for: Evolving brain and behaviour changes in rats following repetitive subconcussive head impacts
Source: Brain Commun. 2023 Nov 20;5(6):fcad316. doi: 10.1093/braincomms/fcad316 (PMC10691880; doi:10.1093/braincomms/fcad316)
Supplement: fcad316_Supplementary_Data [file fcad316_supplementary_data.docx]

**Supplementary materials**

**Evolving brain and behavior changes in rats following repetitive subconcussive head impacts**

Wouter S. Hoogenboom, Todd G. Rubin, Kamalakar Ambadipudi *et al*.

**Supplementary Table 1**. Immunohistochemistry

**Supplementary Figure 1.** Titrating the impact parameters

### Supplementary Figure 2. MRI setup

### Supplementary Figure 3. MRI acquisition, analysis, and regions-of-interest

### Supplementary Figure 4. Behavioral assessment paradigms used

**Supplementary Figure 5**. Histology and immunohistochemical protocols

**Supplementary Figure 6.** Immunohistochemical quantification

Supplementary Figure 7. Weight gain during the impact protocol (P32 to P42) is blunted in female sham and RSHI animals

Supplementary Figure 8. Linear regression for FA ROIs among female groups

Supplementary Figure 9. Linear regression for AD ROIs among female groups

Supplementary Figure 10. Linear regression for MD ROIs among female groups

Supplementary Figure 11. Linear regression for RD ROIs among female groups

Supplementary Figure 12. Linear regression for FA ROIs among male groups

Supplementary Figure 13. Linear regression for AD ROIs among male groups

Supplementary Figure 14. Linear regression for MD ROIs among male groups

Supplementary Figure 15. Linear regression for RD ROIs among male groups

Supplementary Figure 16. Acute rate of change in female DTI metrics, by ROI segments

Supplementary Figure 17. Long-term rate of change in female DTI metrics, by ROI segments

**Supplementary Figure 18.** Foot faults increase as beam narrows

Supplementary Figure 19. H&E-stained sections show no gross morphological abnormalities

**Supplementary Figure 20.** Longitudinal radial diffusivity of the corpus callosum following the final impact.

| Supplementary Table 1. Immunohistochemistry | | | | | | |  |
| --- | --- | --- | --- | --- | --- | --- | --- |
| Primary antibody | Supplier | Catalog # | Conc. | Secondary antibody | Supplier | Catalog # |  |
| Iba-1 | Wako, Richmond, VA | 019-19741 | 1:3000 | HRP anti-rabbit IgG | Vector Laboratories, Burlingame, CA | MP-7451 |  |
| GFAP | BD Biosciences, San Jose, CA | 556330 | 1:100 | HRP anti-mouse IgG | Vector Laboratories, Burlingame, CA | MP-7452 |  |
| MBP | BioLegend, San Diego, CA | SMI-99P | 1:3000 | HRP anti-mouse IgG | Vector Laboratories, Burlingame, CA | MP-7452 |  |
| APP (clone 22C11) | Millipore, Temecula, CA | MAB348 | 1:80 | HRP anti-mouse IgG | Vector Laboratories, Burlingame, CA | MP-7452 |  |
| PHF-1 | Dr. Peter Davies, Feinstein Institute, Manhasset, NY | n/a | 1:500 | Goat anti-mouse IgG1 | SouthernBiotec, Birmingham, AL | 1070-08 |  |
| Abbreviations: Iba-1, ionized calcium binding adapter molecule 1; GFAP, glial fibrillary acidic protein; MBP, myelin basic protein; APP, amyloid precursor protein; PHF-1, paired helical filament-1; HRP, horse radish peroxidase; IgG, immunoglobulin G; Conc., concentration. | | | | | | | |


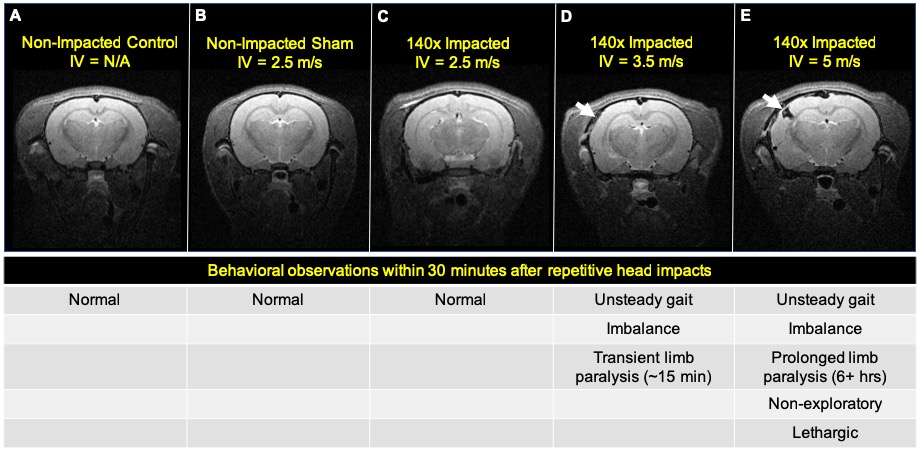


**Supplementary Figure 1.** Titrating the impact parameters

Our choice of impact velocity was designed to fall below that used to produce concussion. We performed a preliminary dose-escalation impacting awake animals (P35) with a range of velocities between 2.5 and 5m/s (20 impacts per day for 1 week) at 5mm “impact depth” and 100ms dwell time (note that in our study, impact depth is the travel of the impactor tip past the position of the surface of the head at time of impact). Animals were carefully observed each day for 30 minutes following head impacts to assess for any overt changes in behavior. 24 hours after final impacts (P42), animals underwent MRI. After careful review by an experienced neuroradiologist (MLL), no evidence of any gross abnormality (i.e., skull fracture, hemorrhage) were visible on T2-weighted images of non-impacted control, sham, and 2.5m/s impacted animals (A-C). Also, rats exhibited regular grooming behavior and ambulated normally. However, animals struck at greater impact velocity (i.e., 3.5 and 5m/s) showed evidence of intracranial hemorrhage and depressed skull fracture (D, E). In addition, we observed various neurological signs and behavioral abnormalities, including imbalance, unsteady gait, transient right forelimb paralysis and non-exploratory, lethargic behavior. These imaging and behavioral features indicative of moderate to severe TBI are inconsistent with subconcussive injury. We therefore selected 2.5m/s impact velocity for the RSHI protocol used in this study.

###
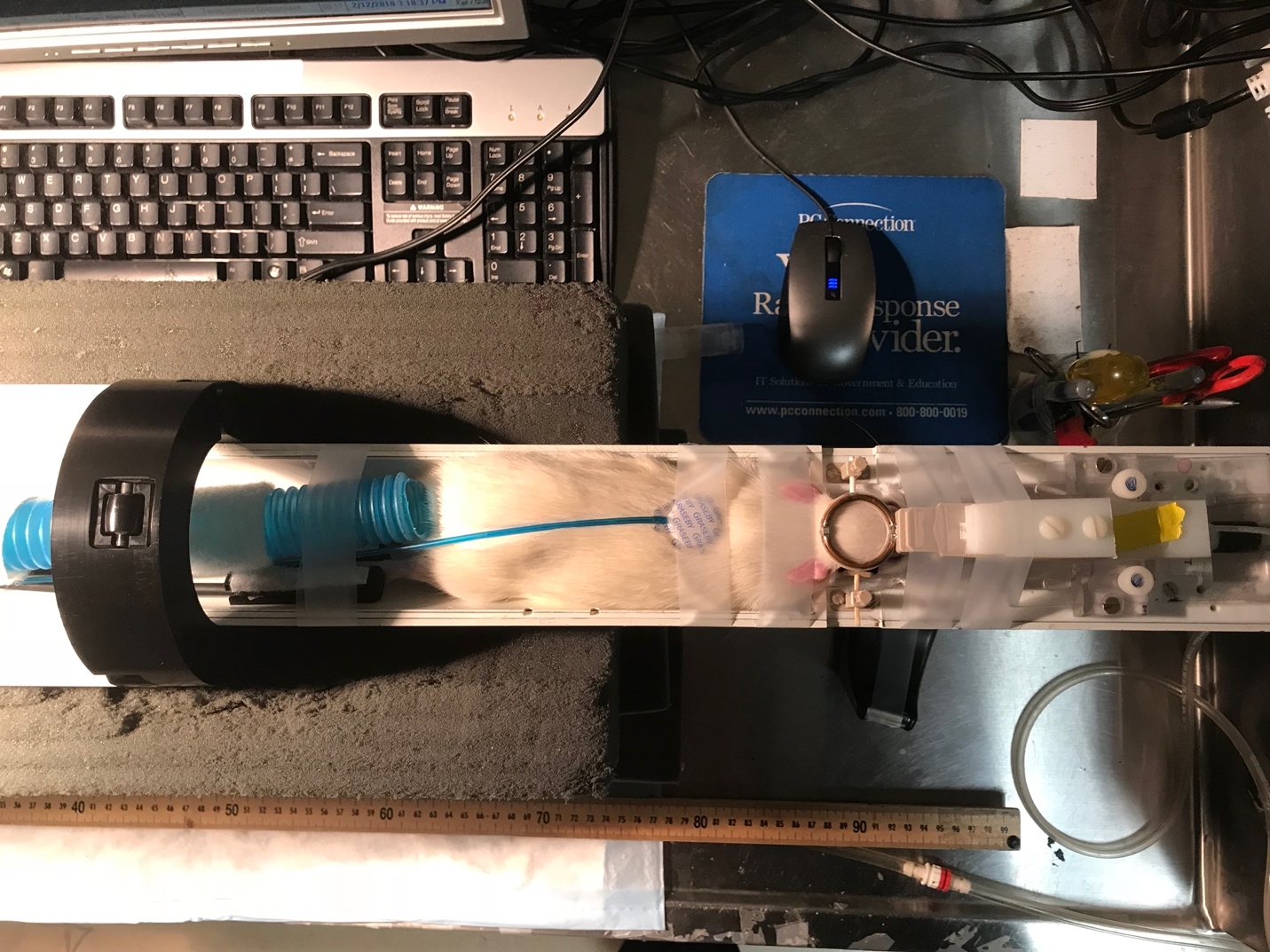


### Supplementary Figure 2. MRI setup

Animals were endotracheally intubated with a 16G catheter, and mechanically respirated at approximately 6 ml/Kg and 66 breaths/min (Small Animal Ventilator Model 683; Harvard Apparatus Co, South Natick, MA) with 1.5-1.75% isoflurane in room air for anesthesia during MRI. Throughout the duration of imaging, warm air was circulated to maintain a body temperature of 38C. Animals were placed in an MRI holder (M2M, Cleveland, OH) with custom 3D printed head restraints. A 20mm receive-only surface coil (Doty Scientific, Columbia, SC) was placed directly onto the rat’s head. Images were acquired using a 9.4T scanner (Agilent Technologies, Wilmington, DE) and a 90mm transmit bird-cage coil (M2M). Great care was taken during animal positioning, and anatomical landmarks were used to ensure consistent placement of acquisition slices.

*
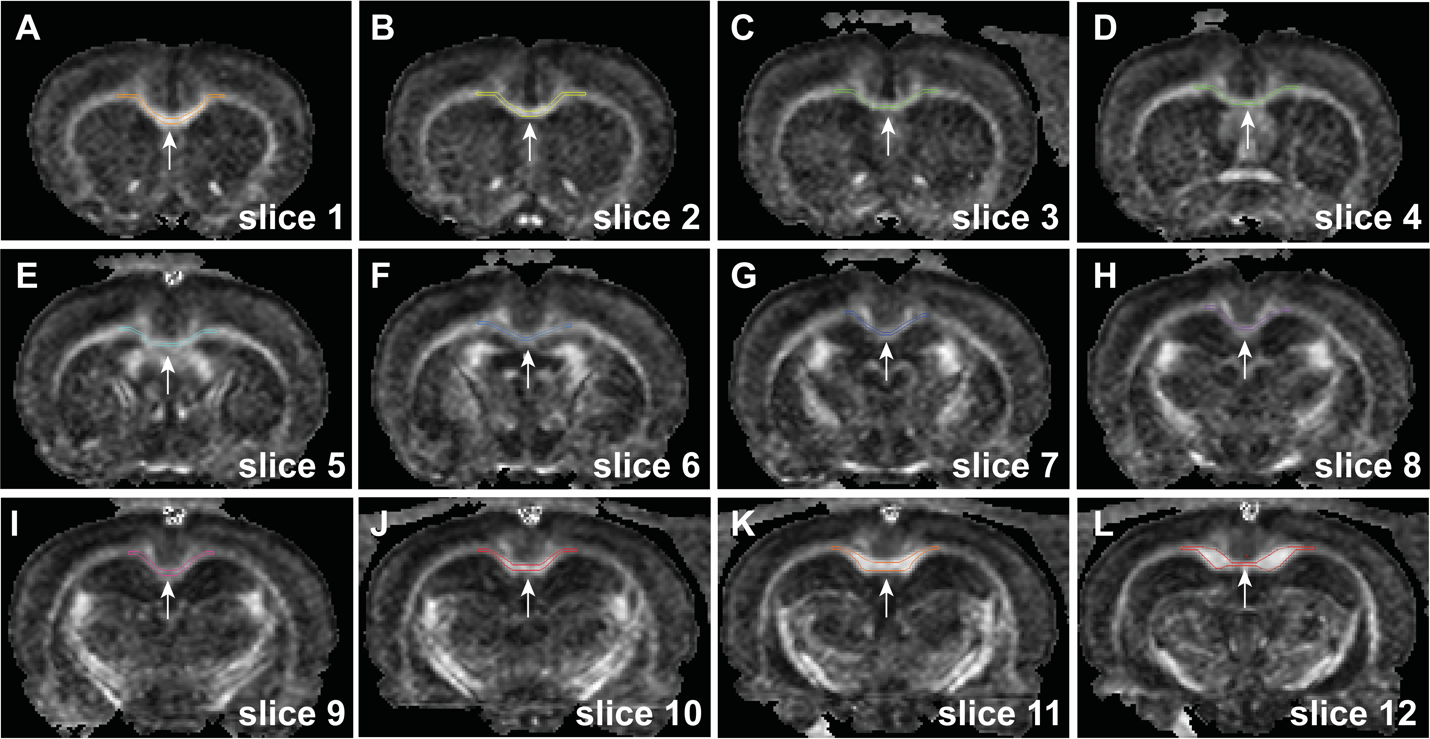
*

### Supplementary Figure 3. MRI acquisition, analysis, and regions-of-interest

DTI was achieved using a spin-echo (SE) echo-planar imaging (EPI) readout with multi-shot k-space acquisitions (8 ‘shots’, echo time 23.87ms, repetition time 4000ms). DTI data were acquired using 30 “electro-repulsive” diffusion directions with *b* = 822 s/mm^2^ and one *b* = 0 s/mm^2^ acquisition, with 3.4ms long diffusion gradients separated by 10ms delta, resolution = 128 × 128, 24-0.5mm interleaved slices an axial plane with 32mm^2^ field of view resulting in images of 0.25 × 0.25 × 0.5 mm voxel size. High resolution T2W images (TR = 5000ms, ESP = 7.97ms, segments = 64, ETL = 4, kzero = 4, effective TE = 31.87ms, averages = 1, repetitions = 1, dummy scans = 2, data matrix = 256x256, FOV = 32mm^2^, slices = 24, thickness = 0.5mm, gap = 0mm, spectral width = 83333Hz) were also acquired to ensure the absence of gross abnormalities such as skull fracture, hemorrhage, and edema. Any animal exhibiting these findings were removed from the study. Scan duration was approximately 60 minutes. Rats were extubated once they were able to move independently and then observed for an additional 30 minutes in room air and a warm incubator before being returned to their home cage. DTI images were eddy current corrected and processed using the FMRIB Diffusion Toolbox^1^ to estimate the diffusion tensor and to compute fractional anisotropy (FA), radial diffusivity (RD), axial diffusivity (AD), and mean diffusivity (MD) at each voxel. Images were deidentified and randomized as to experimental group and acquisition time prior to any analysis. The corpus callosum and bilateral external capsule were manually traced by three trained raters on the *b* = 0 volume using the Medical Imaging Processing and Visualization software package (MIPAV, v8.0.2). Regions-of-interest were selected not only because they are commonly affected in both clinical and animal studies of TBI, but also due to their large volume of white matter, which minimizes partial volume effects.^2^

Corpus callosum and external capsule were manually traced in 215 MRI acquisitions on 12 consecutive coronal slices defined by anatomical boundaries adapted from our previously published study^3^ with minor modifications. Briefly, the corpus callosum was carefully defined by anatomical boundaries on coronal slices guided by the Paxinos and Watson rat brain atlas:^4^ The anterior boundary was the first slice where the corpus callosum crossed at midline. The lateral boundaries were defined at the superior apex of the corpus callosum white matter before it arches back down into external capsule. The posterior boundary was the last slice where the corpus callosum crossed at midline. The corpus callosum (CC) spanned 10 to 14 contiguous coronal slices (mean = 12 slices). The ipsilateral external capsule (IEC) was traced in the left hemisphere (impact side) on every slice containing CC starting at the lateral boundary of the CC continuing to the apex of the most lateral white matter. The contralateral external capsule was traced similar to ipsilateral external capsule, but in the right hemisphere (non-impact side).

###
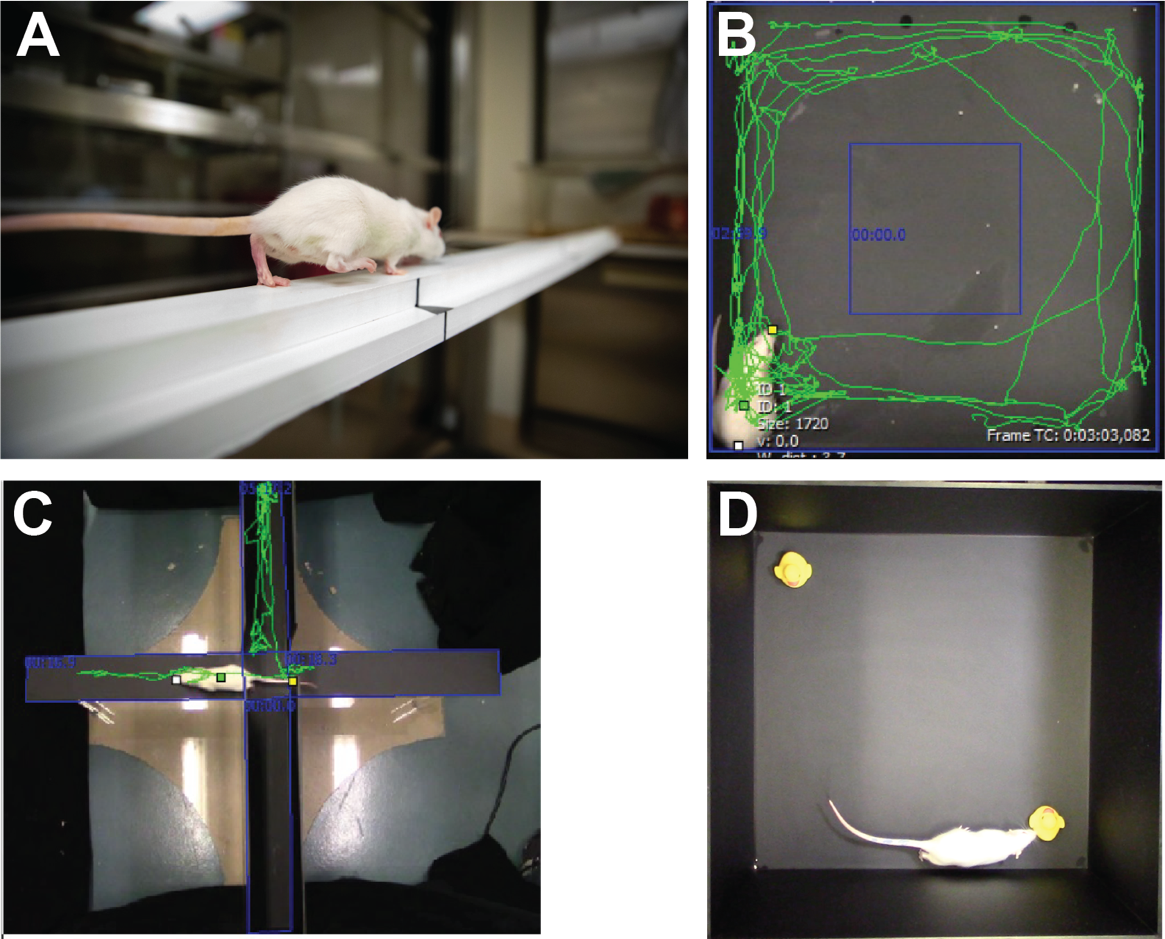


### Supplementary Figure 4. Behavioral assessment paradigms used

All equipment used for behavioral testing was in-house constructed of non-reflective, non-porous and opaque acrylic materials (Plastic-Craft Products, West Nyack, NY). Every assessment was recorded with a high-definition video camera (Canon VIXIA HF R800 Camcorder; 1920x1080 pixels at 60 frames per second) on a stable video tripod (Magnus VT-200) and scored by trained observers blind to animal or group ID (beam walking, novel object placement) or using automated tracking software (open field, elevated plus maze) using a three-point detection algorithm that enabled identification of the animal’s nose tip, center of gravity and tail base (Biobserve Viewer 3, Germany). Equipment was cleaned with 70% ethanol between animals and at the end of each day of testing.

(**A**) The beam balance (BB) test was used to assess motor coordination^5, 6^ that involves scoring foot faults (slips) during traverse of the beam. We used a 165cm long tapered beam as described and validated previously in both aged and young adult rats^7, 8^ The beam, located approximately 1m above the floor, consisted of a 15cm untapered loading zone at the beginning, three tapered 45cm intervals (interval 1-3) along the beam to enable scoring of the foot fault’s location, and a 15cm untapered unloading zone at the end. The animal’s home cage was placed at the end of the beam as a reinforcer. A 2cm wide ledge was present along each side of the beam, 2cm below the upper surface of the beam, to prevent falling in the event of a foot fault. Animals were tested every day immediately after the impact protocol as well as 24 hours and 4 weeks after the final day of impacts. Animals were allowed to walk the beam for a maximum of 6 minutes, or until completing 5 full crossings, whichever came first. The number of beam crossings was recorded, as well as the number of foot faults for each section of the beam. In case animals did not cross completely, beam crossings were scored with partial points (interval 1 = 0.25 beam crossing, interval 2 = 0.5 beam crossing interval 3 = 0.75 beam crossing). Animals that turned around and started walking in backward direction were picked up and returned to the last forward moving location before turning around. Only forward steps were recorded. The total distance walked on the beam was calculated as number of beam crossings multiplied by 165cm (length of the beam). Foot faults on the BB were corrected for distance and reported as percentage faults per 100cm travelled on the beam.

(**B**) The open field (OF) is one of the most widely used measures of behavior in animal studies, which has been validated in adolescent rats.^9^ The OF is generally used to assess locomotor activity, anxiety, response to novelty stress and exploration.^10^ Animals were assessed at 24 hours and 4 weeks after the final day of impacts. The animal was placed in a square, open box (50cm length x 50 cm width x 35cm height) and allowed to explore the arena for 3 minutes during which time locomotion (track length) and thigmotaxis (exploration of the periphery vs. the center) were recorded and scored by automated tracking software.

(**C**) The elevated plus maze (EPM) is a widely used test to assess anxiety-related behavior in rodents, which has been validated in adolescent rats.^9^ The equipment setup has been previously described.^11^ In short, the structure has four arms (50cm long and 10cm wide) two of which are enclosed (30cm high walls) and two open without any wall or railing. Each arm was supported by sturdy plastic legs that maintain the arms 50cm above the floor. The EPM assesses preference between a comparatively safe environment (the closed arms) and a risky environment (open arms). The general principle is that the more anxious the animal, the less likely it will explore an uncomfortable, risky or threatening environment. Animals were assessed at 24 hours and 4 weeks after the final day of impacts. The animal was placed in the center of the EPM to start and allowed to explore for 6 minutes during which time locomotion (track length) and thigmotaxis (exploration of the open vs. closed arms) were recorded and scored by automated tracking software.

(**D**) The novel object placement (NOP) test is a test of spatial memory and is based on the rodent’s robust preferential exploration of novel or displaced objects. Animals were assessed at 24 hours and 4 weeks after the final day of impacts. The animal was placed in the arena (same box used for the OF paradigm), which contained 2 identical objects (vinyl yellow duck bath toys, width = 1.875in, height = 2in, depth = 2.25in, Dollar Tree, Inc., SKU#259523), and allowed to explore for 3 minutes (sample trial 1). In the subsequent test trial (trial 2), with a retention interval of 1 hour, one of the objects was displaced in space according to a randomized design - that is, the objects were randomized in terms of trial 1 placement and subsequent placement of the moved object to ensure location was not affecting preference. In both trials, the time exploring each object was recorded by a trained rater.

**
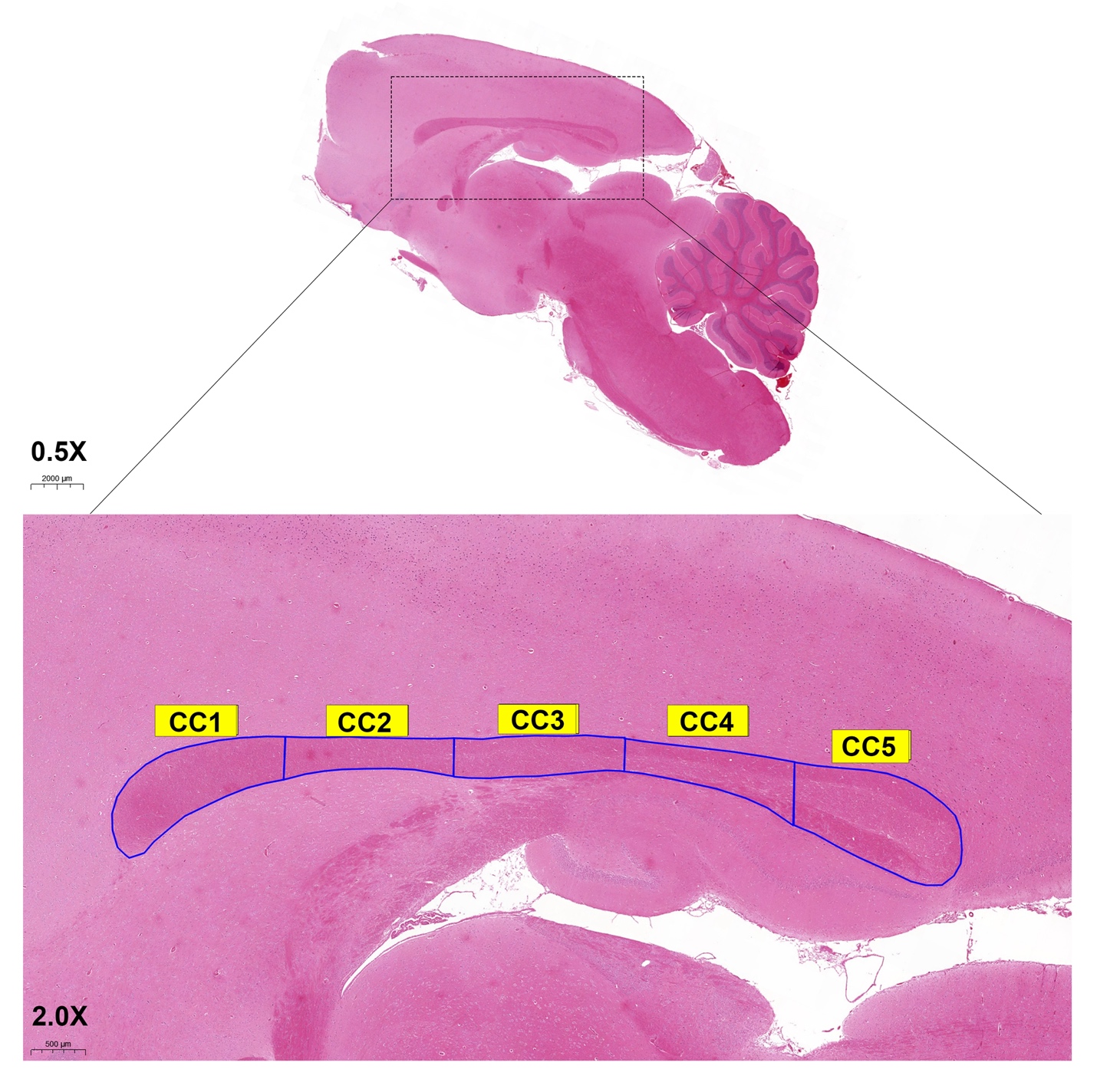
**

**Supplementary Figure 5**. Histology and immunohistochemical protocols

Thickness of the corpus callosum (CC) was measured on hematoxylin and eosin (H&E) de-identified digitized images at 11 locations 864µm apart along the rostro-caudal dimension of the CC (see black arrows). Average thickness was calculated for the whole CC and for 5 segments (CC1 = genu, CC2 = anterior mid-body, CC3 = mid-body, CC4 = posterior mid-body, and CC5 = splenium). All histological and immunohistochemical (IHC) procedures were performed at Albert Einstein College of Medicine, Histotechnology and Comparative Pathology Facility, except for amyloid precursor protein (APP) and Bielschowsky silver stain, which were performed by Histoserve (https://www.histoservinc.com).

### H&E stain

First, paraffin sections were thoroughly de-waxed with xylene in a two-step process (8 and 7 minutes). The slide was then passed through several changes of alcohol (2x100% alcohol for 3 minutes, 95% alcohol for 2min, 80% alcohol for 2min, 75% alcohol for 1min) to remove the xylene, and then thoroughly rinsed in water (2 min) to hydrate the tissue so that aqueous reagents could readily penetrate the cells and tissue elements. The slide was then stained with hematoxylin (Poly Scientific R&D Corp, catalog # s212) for 8 min and washed twice (10s and 5min, respectively). To remove non-specific background staining and to improve contrast, the tissue was shortly exposed (5s) to a weak acid alcohol (1% acid alcohol: 6ml HCl [12.5N] in 600ml of 70% alcohol), followed by a 3min wash, 10s ammonia (ammonia water: 1.5ml of 28% ammonium hydro in 600ml tap water), 4min wash, and 4min 80% alcohol. The eosin counterstain was then applied for 8min. Following the eosin stain, the slide was passed through three 1min changes of 100% alcohol to remove all traces of water, and then rinsed in three baths of xylene (2, 5, and 5min respectively) to clear the tissue and to render it completely transparent, and finally cover slipped.

Bielschowsky silver stain

The unstained slides were deparaffinized in xylene, then hydrated through graded alcohols to water, then rinsed well in distilled water. The slides were then placed in silver nitrate in the dark at 37⁰C. The slides were then transferred to distilled water and ammonium hydroxide was added to the silver solution. Once the ammoniacal silver was prepared, the slides were put in the ammoniacal silver solution in the dark at 37⁰C.  The slides were then put in ammonium hydroxide and a developing solution was added to the ammoniacal silver. The slides were transferred back to the ammoniacal silver/developing solution and agitated until black plaques were visible on the slides. The slides were then washed in ammonium hydroxide, washed in sodium thiosulfate, then washed in distilled water. The slides were then dehydrated through graded alcohols, cleared in xylene, and cover slipped with permount.

The immunohistochemical (IHC) protocol steps were as follows (see Supplementary Table 1 for all primary and secondary antibodies used in this study): First, sections were pre-heated at 60°C for 60min and then de-waxed with xylene (2x10 minutes) followed by several alcohol washes (100% alcohol, 2 x 10min; 95% alcohol, 2 x 2 min; 80% alcohol, 2 x 2 min; 70% alcohol, 2 x 2 min). Sections were then washed in TBS buffer (2 x 2min) and blocked with 3% H_2_O_2_ for 20min at room temperature followed by antigen retrieval using 10mM pH 6.0 citrate buffer steamed for 20min. After 30min cooling, tissues were thoroughly washed in TBS for 2 x 3min and then blocked in 5% goat serum/2% BSA for 30min at room temperature. Primary antibody was added to the blocking solution and incubated for 60min at room temperature. After 3 additional washes in TBS, secondary antibody was applied and then washed 2 x 5min. Staining was visualized with diaminobenzidine (DAB) applied for 3min. Following 30s counter stain with 1/3 diluted Harris hematoxylin, slides were dehydrated and cover slipped with xylene-based mountant.

Paired helical filament (PHF-1) (generously provided by Dr. Peter Davies, Feinstein Institute/Northwell, NY) is a mouse monoclonal antibody specific for phosphorylation sites S396 and S404 in tau.^12, 13^ Sections were incubated in 3% hydrogen peroxide/0.25% Triton X100 for 30min followed by incubation in 5% milk in TBS for 1h at room temperature. Primary antibody, PHF1, was added and diluted in 5% milk in TBS overnight in the cold room, and then washed 4 times for 5min each in TBS+0.05% Triton X100 at room temperature. Secondary antibody, goat anti-mouse IgG1 biotin labeled was added, diluted 1:500 in 20% Superblock (ThermoFisher, catalog # 37535) diluted in TBS+0.05% Triton X100 and incubated for 2h at room temp. Sections were washed 4 times for 5min in TBS+0.05% Triton X100 and then incubated for 1h at room temperature in streptavidin-horse radish peroxidase (SouthernBiotech, catalog#7100-05) diluted 1:500 in 20% Superblock diluted in TBS+0.05% Triton X100. Sections were washed 3 additional times for 5min in TBS+0.05% Triton X100 and then visualized with 0.3mg/ml of DAB in 100mM Tris, pH 7.4, containing 60 microliters of 30% hydrogen peroxide in 100ml (8 min). Tissues were transferred to TBS, mounted on slides, allowed to dry for 30min, dehydrated and finally cover slipped.


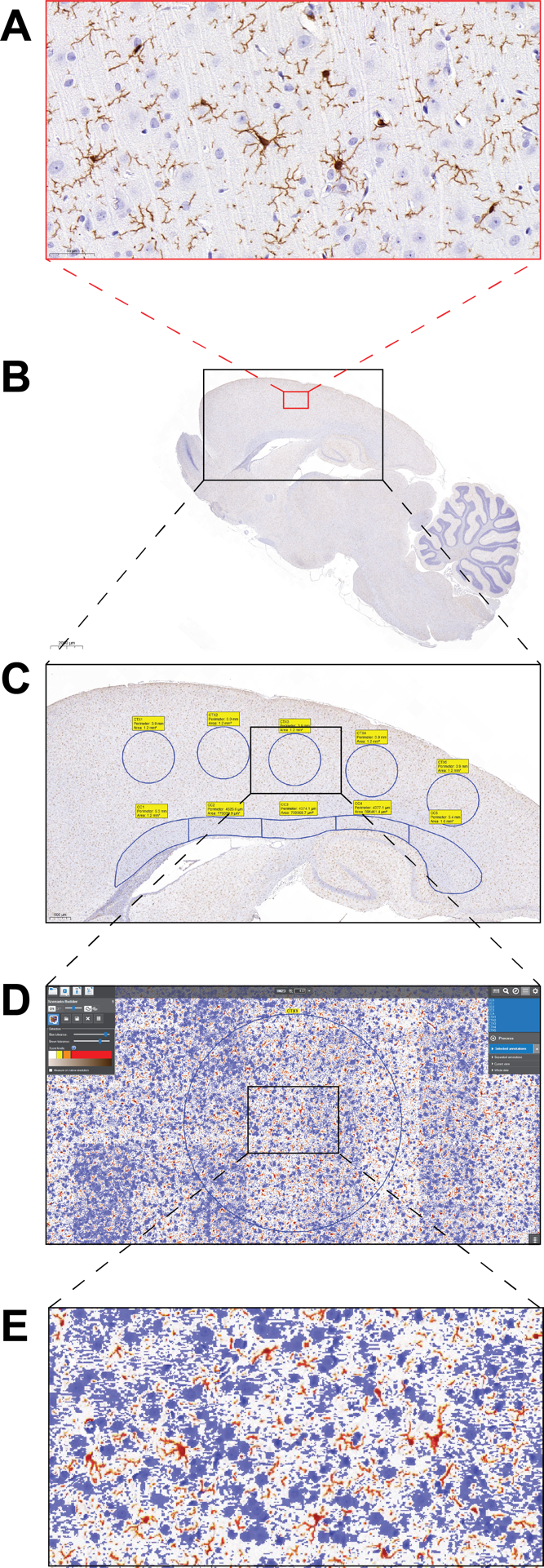


CC5

CC4

CC3

CC2

CC1

CTX5

CTX4

CTX2

CTX3

CTX1

**Supplementary Figure 6.** Immunohistochemical quantification

A digital slide scanner (Pannoramic 250 Flash II, 3DHISTECH Ltd., Budapest, Hungary) was used to obtain whole slide, high-quality digital images (bright-field resolution=41x). All digitized slides passed quality control for visible out-of-focus areas or misalignments. Regions-of-interest (ROIs) were manually delineated by an experienced tracer on de-identified digitized images using CaseViewer software (version 2.2) (3DHISTECH Ltd., Budapest, Hungary). Corpus callosum (CC) was segmented into 5 sections of 1728 ± 128µm in length yielding the following areas: CC1 = genu (surface area=1.12 ± 0.22mm^2^), CC2 = anterior mid-body (surface area = 0.76 ± 0.19mm^2^), CC3 = mid-body (surface area = 0.69 ± 0.13mm^2^), CC4 = posterior mid-body (surface area = 0.47 ± 0.10mm^2^) and CC5 = splenium (surface area = 1.43 ± 0.38mm^2^). The corpus callosum was chosen as it is the largest white matter tract in the brain and clinical data emphasized its vulnerability following pediatric TBI.^14^ Additionally, 5 circular ROIs (surface area = 1.20mm^2^ each) were placed in the cerebral white matter (CTX) halfway between the CC and surface of the brain.

All tissue samples were quantified with an automated approach using the commercially available QuantCenter / DensitoQuant plug-in for CaseViewer (3DHISTECH Ltd., Budapest, Hungary). **Figures A-E** are from an Iba-1-stained section: **A** is a 35x image with visible stained microglia, **B** is a whole brain Iba-1-stained section at 0.5x, **C** has corpus callosum and cerebral white matter ROIs at 1.6x, **Figures D-E** have been analyzed with the QuantCenter / DensitoQuant plug-in. The application is a stain-intensity-based IHC quantification tool that identifies the positive stain based on an automatic color separation method through which individual positive pixels are counted and classified based on intensity and threshold ranges. Blue, white, yellow, orange and red pixels are categorized as background, negative, weak-positive, moderate positive and strong positive pixels, respectively. Brown-stained microglia appear in orange/red color (**D, E**).

Quantification method:

Total pixels = background pixels + negative pixels + weak-positive pixels + moderate-positive pixels + strong-positive pixels

Positive pixels = moderate-positive + strong-positive pixels

% Positive pixels = (positive pixels / total pixels) *100%

Special thanks to Hillary Guzik and Andrea Briceno of the Analytical Imaging Facility at Einstein for their technical assistance with the IHC analysis. Grant support by the Albert Einstein Cancer Center Support Grant of the National Institutes of Health under award number P30CA013330. This work utilized high-speed/resolution whole slide scanner (Pannoramic 250 Flash II) that was purchased with funding from a National Institutes of Health SIG grant 1S10OD019961-01.


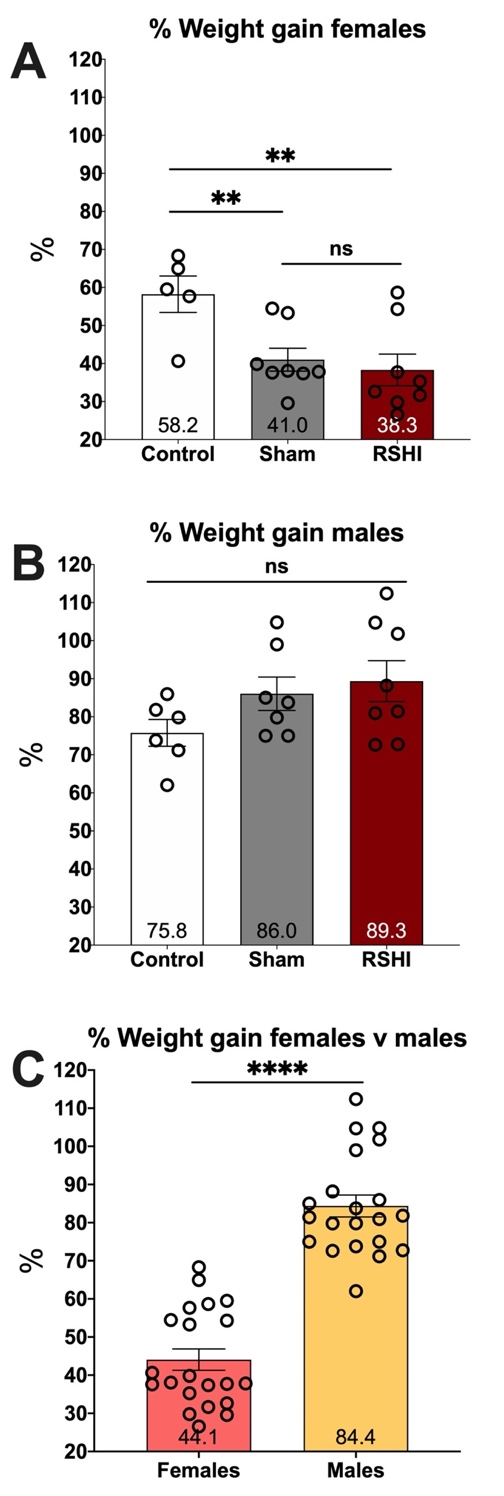


Supplementary Figure 7. Weight gain during the impact protocol (P32 to P42) is blunted in female sham and RSHI animals.

Both female sham (+41.0%) and female RSHI (+38.3%) gained weight during the impact protocol, from postnatal day 32 (P32) to postnatal day 42 (P42), but at a lesser rate than female controls (+58.2%), possibly due to stress associated with daily restraint (**A**). Male groups exhibited equal weight gain (**B**). Overall, male rats (+84.4%) gained relatively more weight than female rats (+44.1%) during the impact protocol (**C**). All data are presented as mean ± standard error of the mean (SEM). ***P* < 0.01, *****P* < 0.0001.


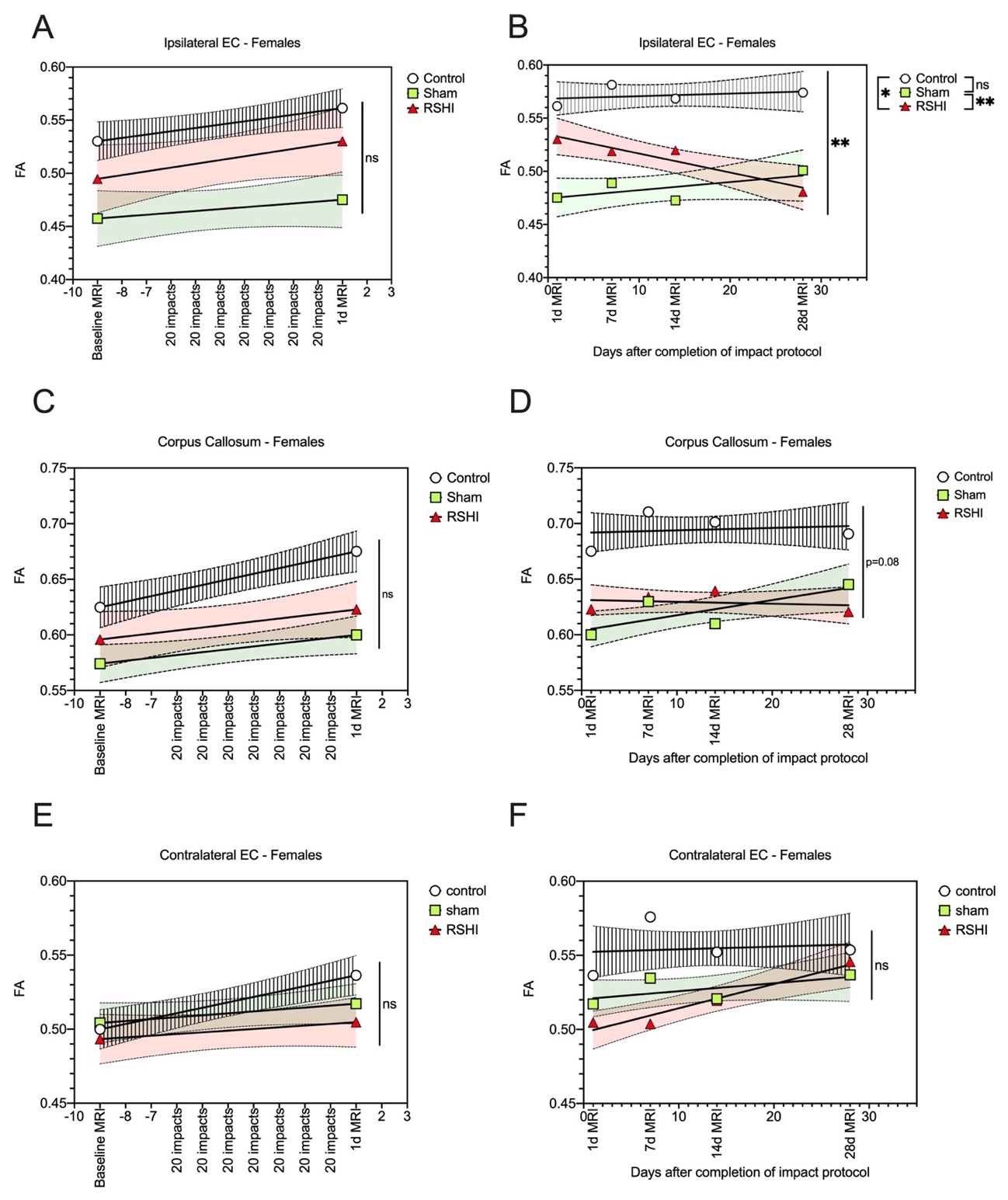


Supplementary Figure 8. Linear regression for FA ROIs among female groups

Significant long-term group differences were observed for the ipsilateral EC (ANOVA *P* = 0.0034) (**B**). Post-hoc analysis with Holm-Sidak’s multiple comparisons test revealed that RSHI animals exhibited FA decline (slope = -0.001784) that significantly diverged from sham (slope = 0.0007631; RSHI v sham, *P* = 0.0041) and control animals (slope = 0.0002386; RSHI v control, *P* = 0.0377). Control and sham animals exhibited equal trends (*P* = 0.5472). Each symbol (white = controls, green = sham, red = RSHI) represents the group mean for that MRI assessment. Black straight lines are best fit regression lines with 95% CI. **P* < 0.05, ***P* < 0.01, ****P* < 0.001. EC = external capsule, FA = fractional anisotropy.


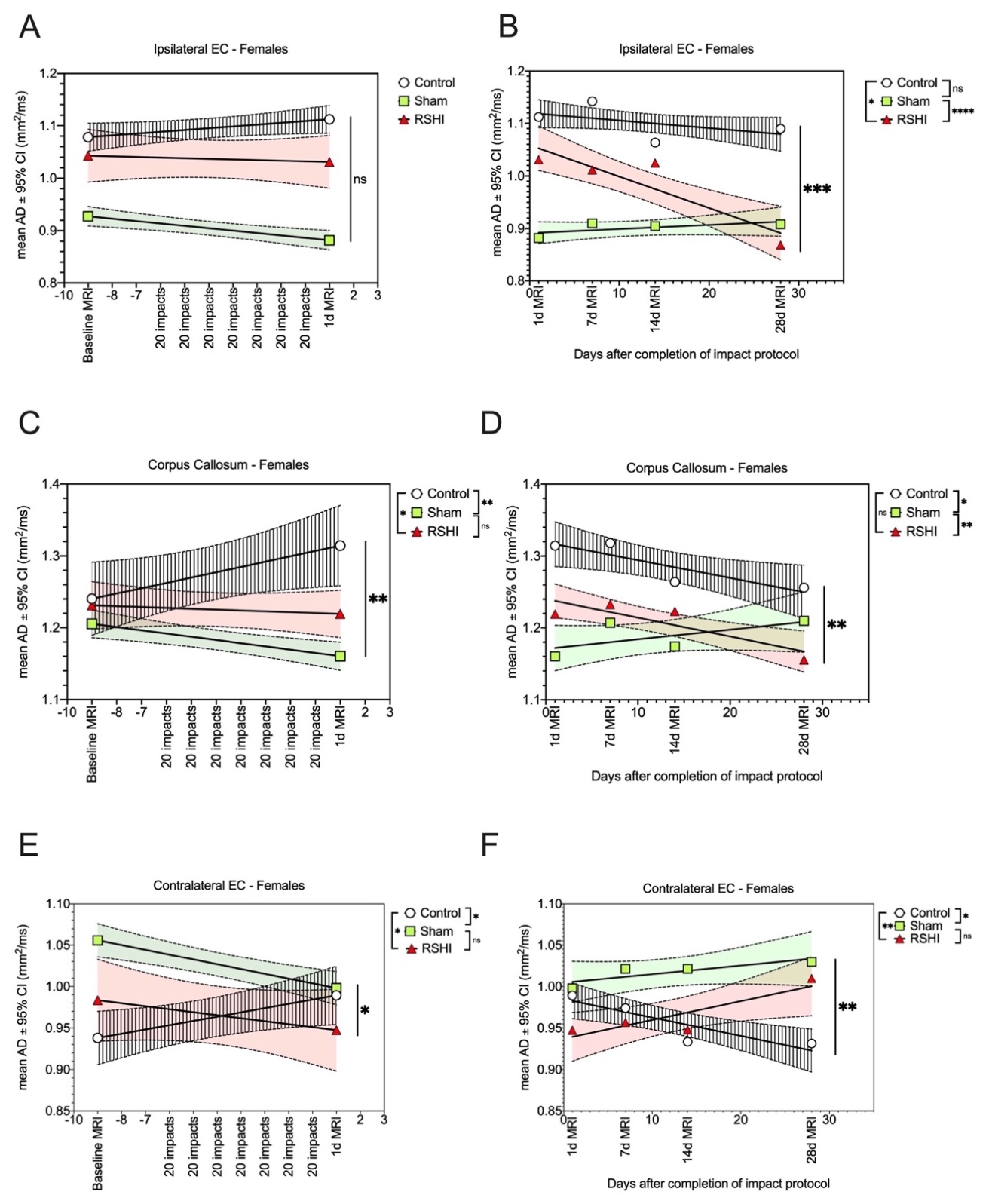


Supplementary Figure 9. Linear regression for AD ROIs among female groups

Significant group differences were observed for every ROI, except acute IEC. During the acute injury response, both sham and RSHI animals exhibited negative DTI trajectories, which diverged from controls in CC and CEC ROIs. As sham animals bounced back in the following weeks, RSHI animals continued to have decreasing AD trajectories for IEC and CC ROIs, but not CEC. Each symbol (white = controls, green = sham, red = RSHI) represents the group mean for that MRI assessment. Black straight lines are best fit regression lines with 95% CI. **P* < 0.05, ***P* < 0.01, ****P* < 0.001. AD = axial diffusivity, EC = external capsule.


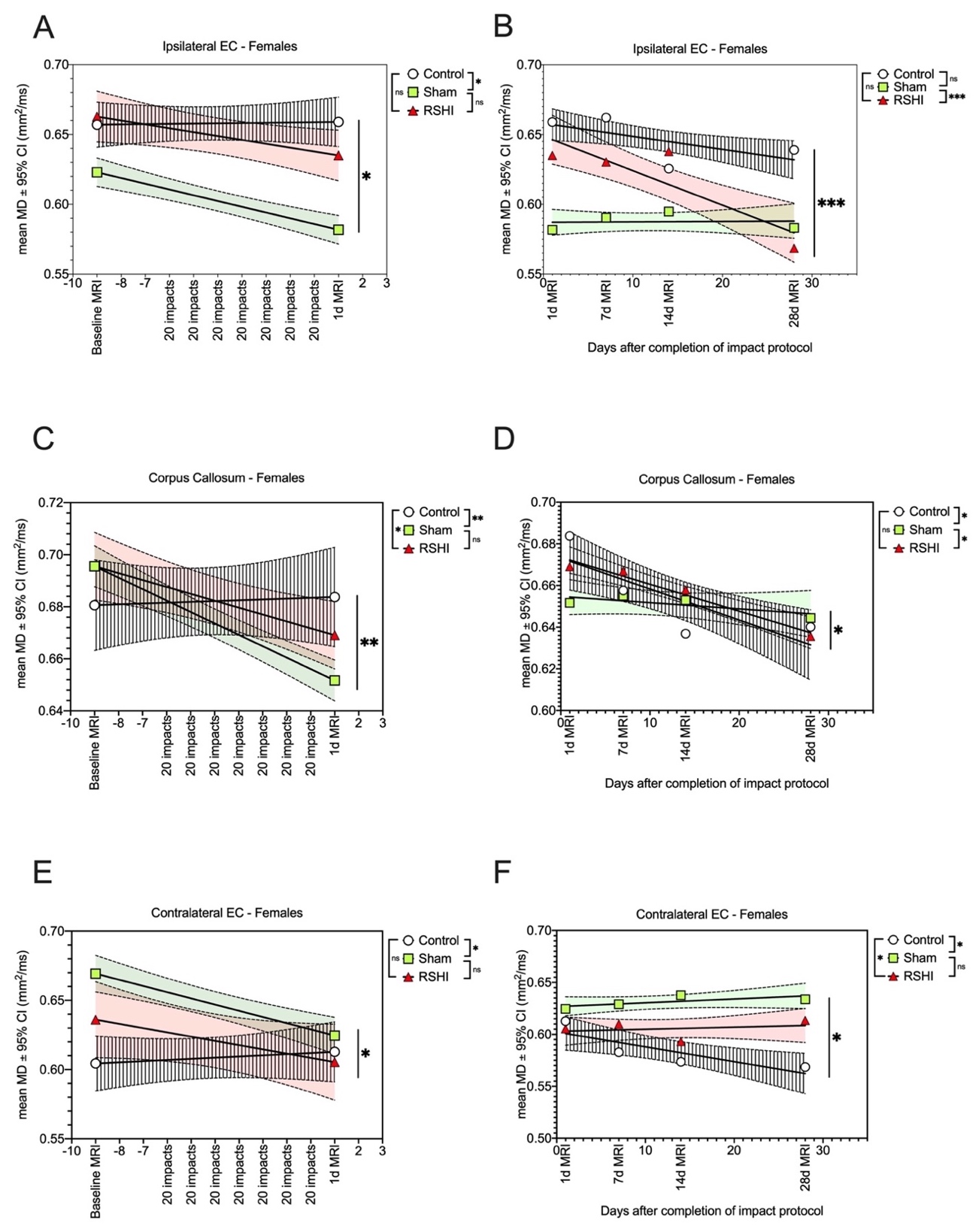


Supplementary Figure 10. Linear regression for MD ROIs among female groups

Significant group differences were observed for every ROI. During the acute injury response, both sham and RSHI animals exhibited negative DTI trajectories, which diverged from controls. As sham animals bounced back in the following weeks, RSHI animals continued to have decreasing MD trajectories for IEC and CC ROIs, but not CEC. Each symbol (white = controls, green = sham, red = RSHI) represents the group mean for that MRI assessment. Black straight lines are best fit regression lines with 95% CI. **P* < 0.05, ***P* < 0.01, ****P* < 0.001. CC = corpus callosum, EC = external capsule, MD = mean diffusivity.


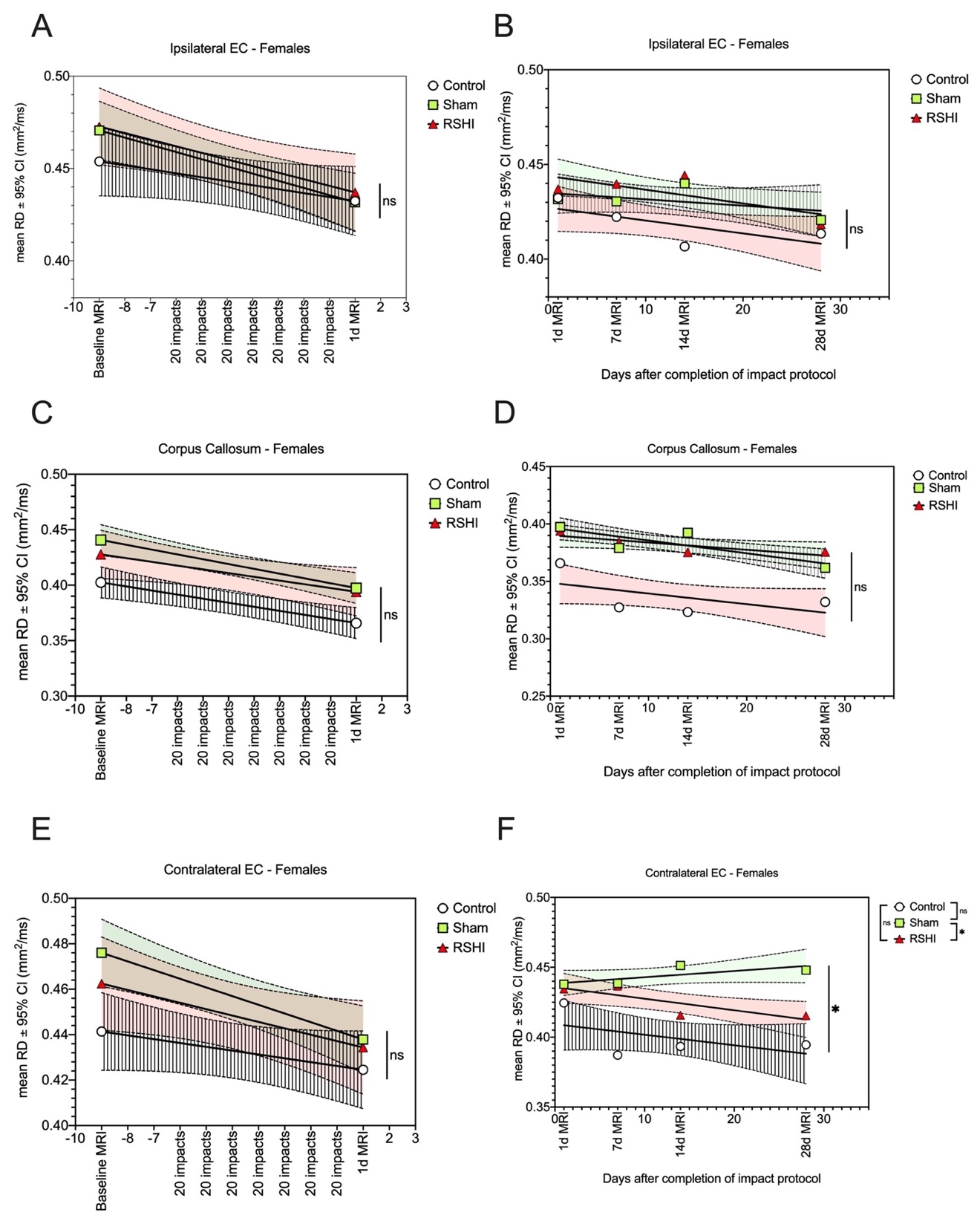


Supplementary Figure 11. Linear regression for RD ROIs among female groups

Significant group differences were only observed for long-term CEC RD (**F**). RSHI and sham had opposite slopes (*P* = 0.045). Each symbol (white = controls, green = sham, red = RSHI) represents the group mean for that MRI assessment. Black straight lines are best fit regression lines with 95% CI. **P* < 0.05, ***P* < 0.01, ****P* < 0.001. EC = external capsule, RD = radial diffusivity.


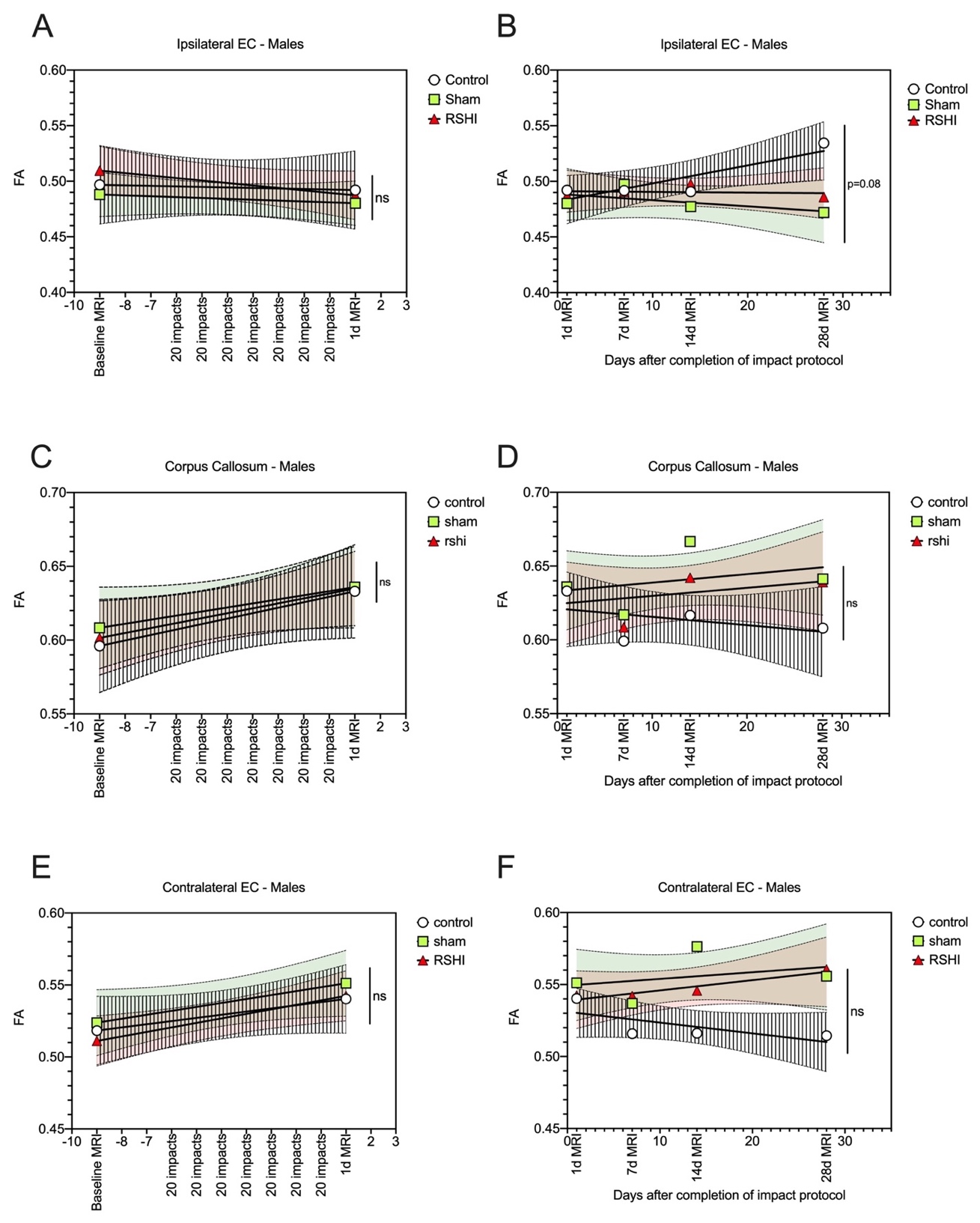


Supplementary Figure 12. Linear regression for FA ROIs among male groups

Each symbol (white = controls, green = sham, red = RSHI) represents the group mean for that MRI assessment. There were no significant group differences. FA = fractional anisotropy, EC = external capsule.


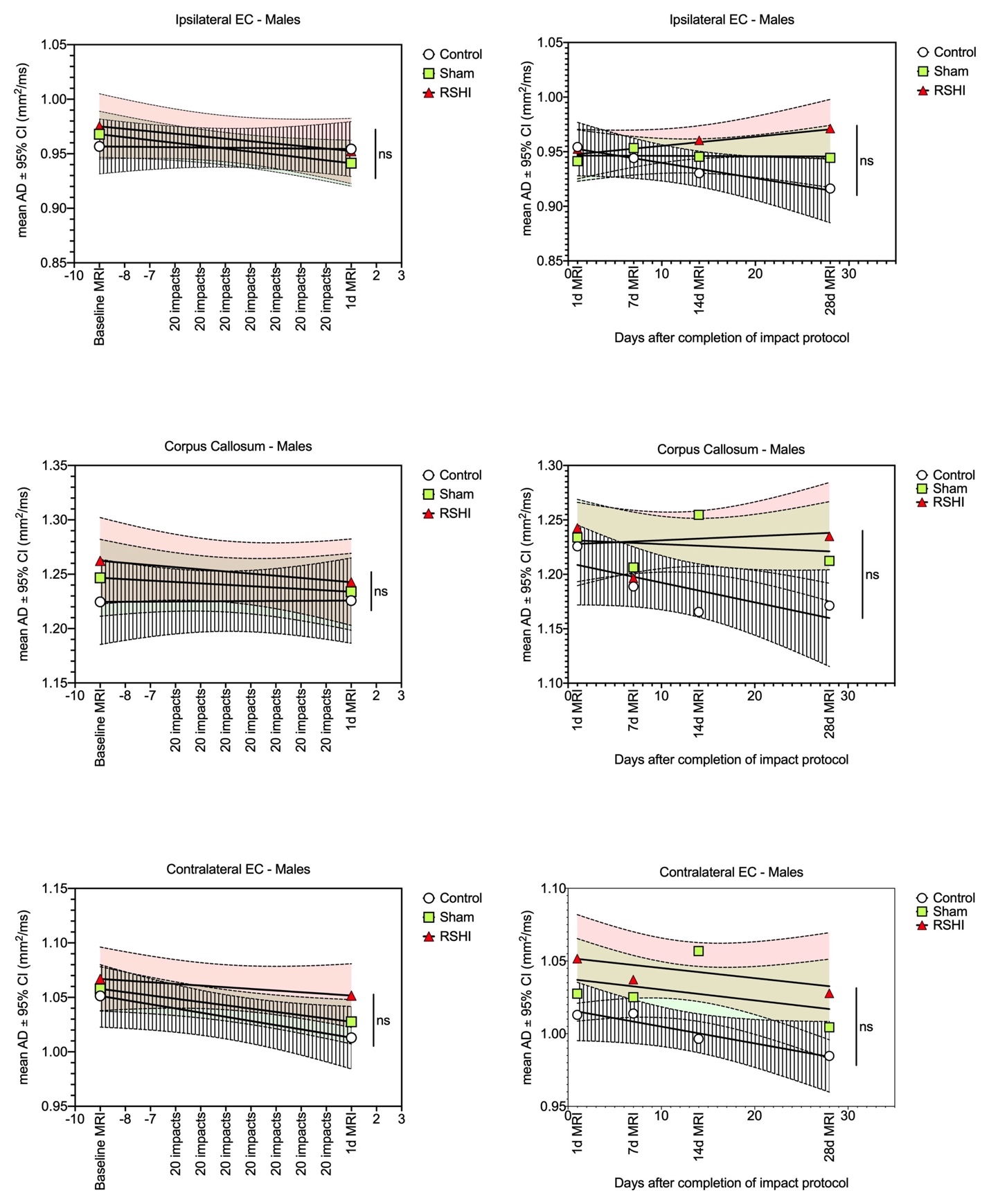


Supplementary Figure 13. Linear regression for AD ROIs among male groups

Each symbol (white = controls, green = sham, red = RSHI) represents the group mean for that MRI assessment. There were no significant group differences. AD = axial diffusivity, EC = external capsule.


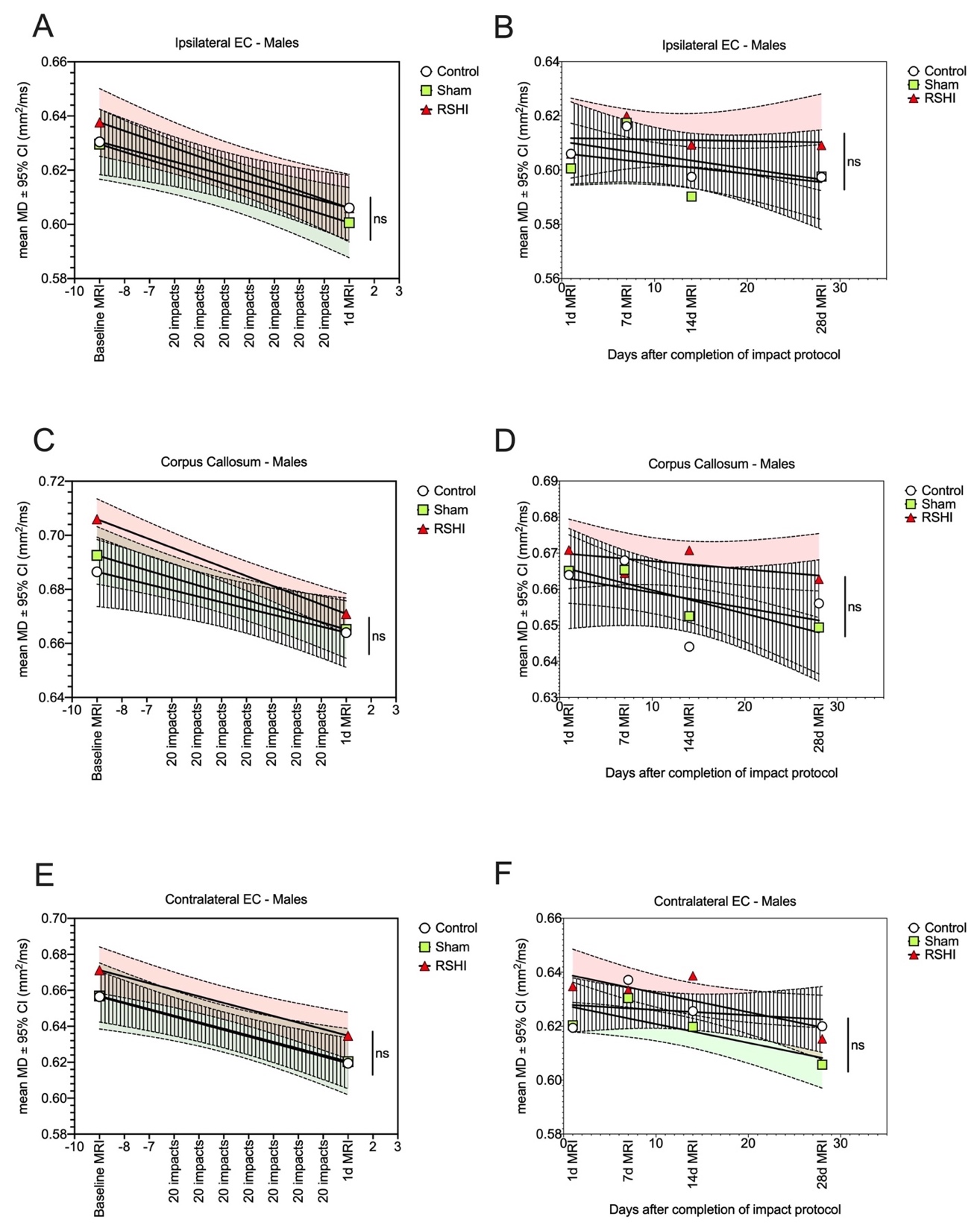


Supplementary Figure 14. Linear regression for MD ROIs among male groups

Each symbol (white = controls, green = sham, red = RSHI) represents the group mean for that MRI assessment. There were no significant group differences. EC = external capsule, MD = mean diffusivity.


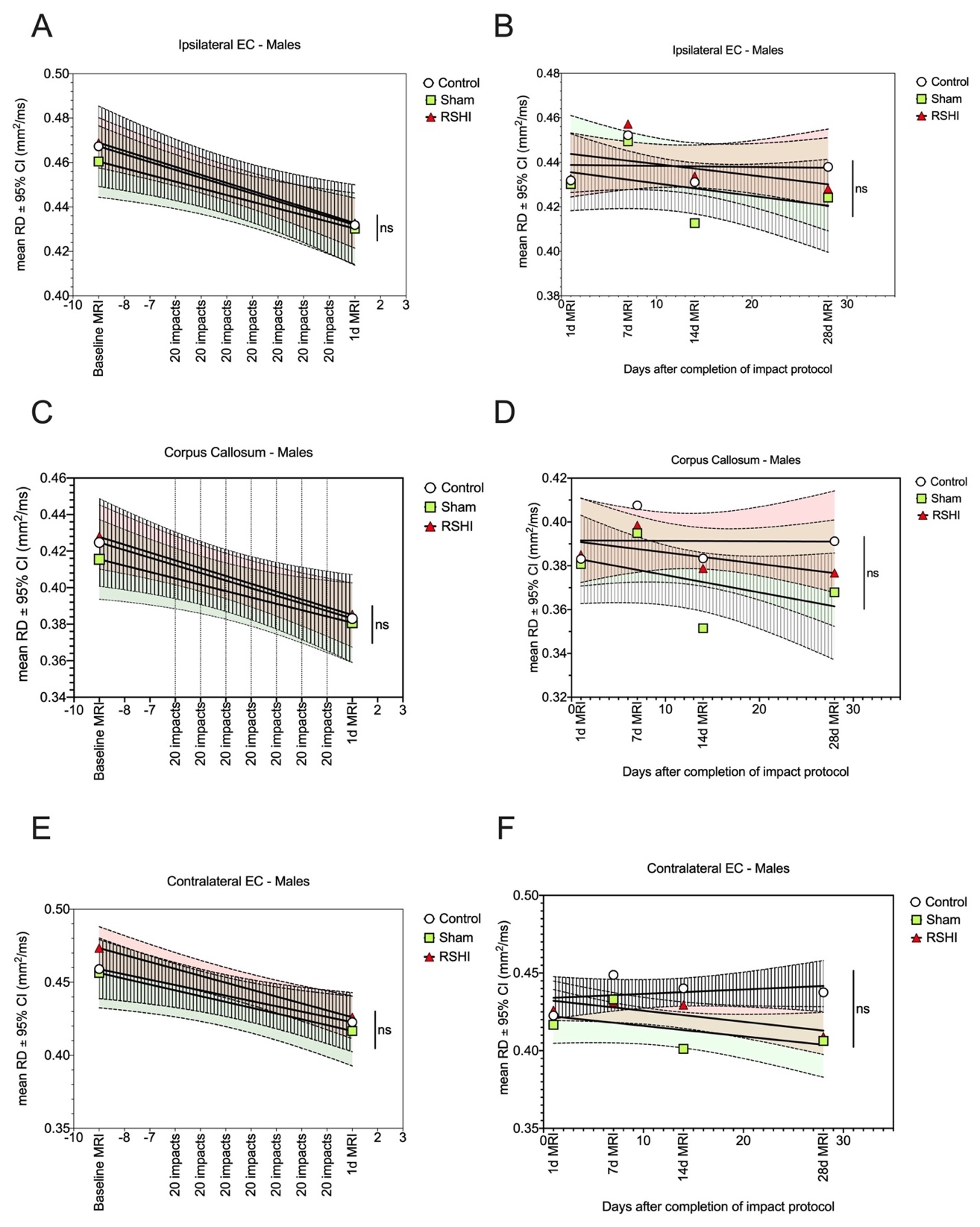


Supplementary Figure 15. Linear regression for RD ROIs among male groups

Each symbol (white = controls, green = sham, red = RSHI) represents the group mean for that MRI assessment. There were no significant group differences. EC = external capsule, RD = radial diffusivity.


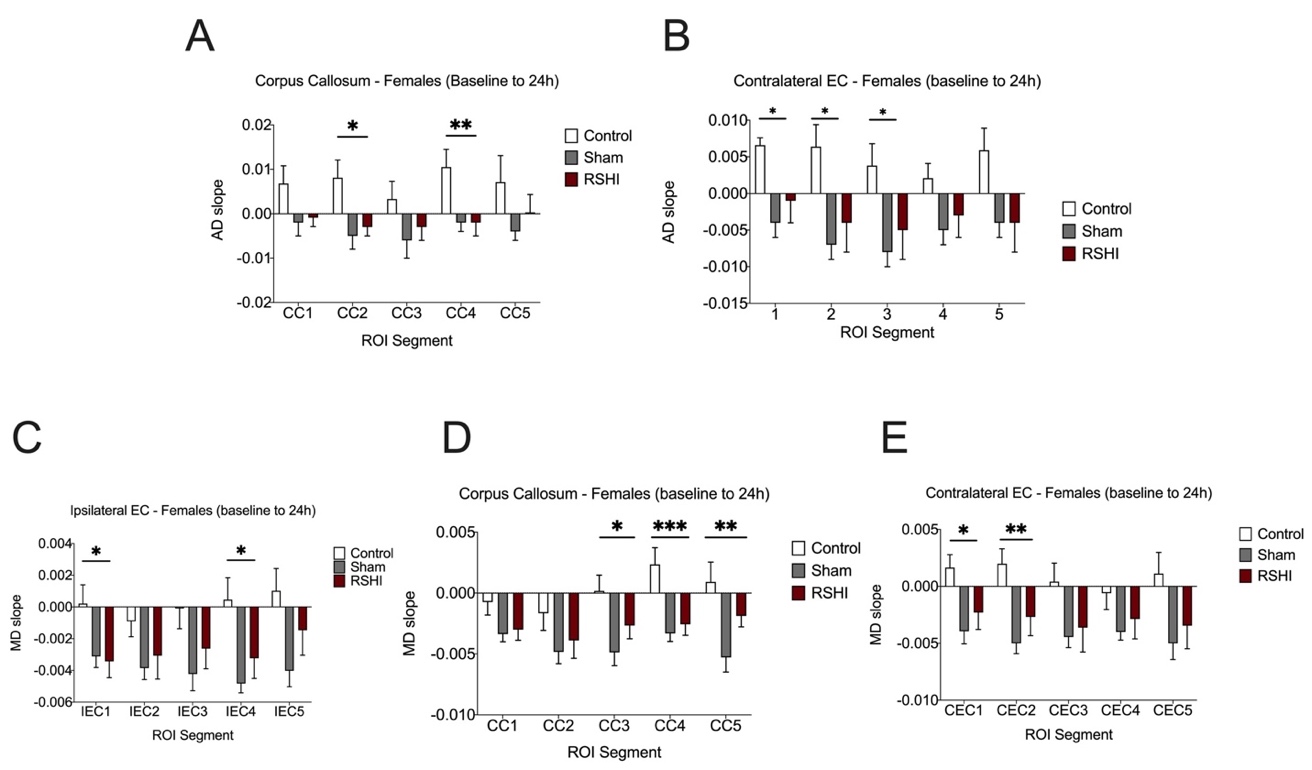


Supplementary Figure 16. Acute rate of change in female DTI metrics, by ROI segments

Controls exhibit positive trajectories opposed to negative trajectories in sham and RSHI. **P* < 0.05, ***P* < 0.01, ****P* < 0.001. AD = axial diffusivity, EC = external capsule, MD = mean diffusivity.


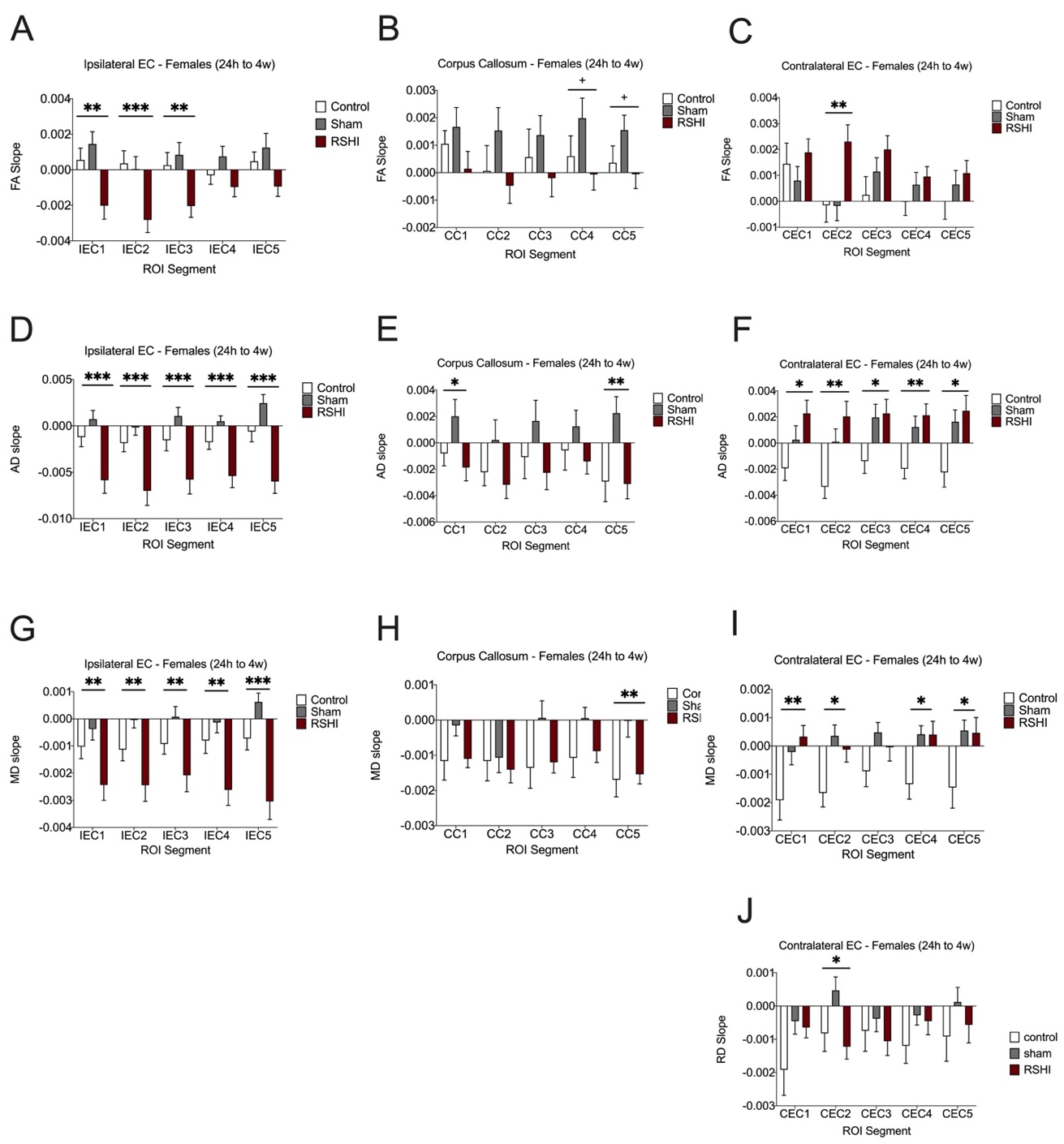


Supplementary Figure 17. Long-term rate of change in female DTI metrics, by ROI segments

RSHI animals’ developmental trajectories diverge from the other groups mostly in the ipsilateral ROIs and anterior mid-body (segment 2) with diffuse and strongly negative trends. **P* < 0.05, ***P* < 0.01, ****P* < 0.001. AD = axial diffusivity, EC = external capsule, FA = fractional anisotropy, MD = mean diffusivity, RD = radial diffusivity.


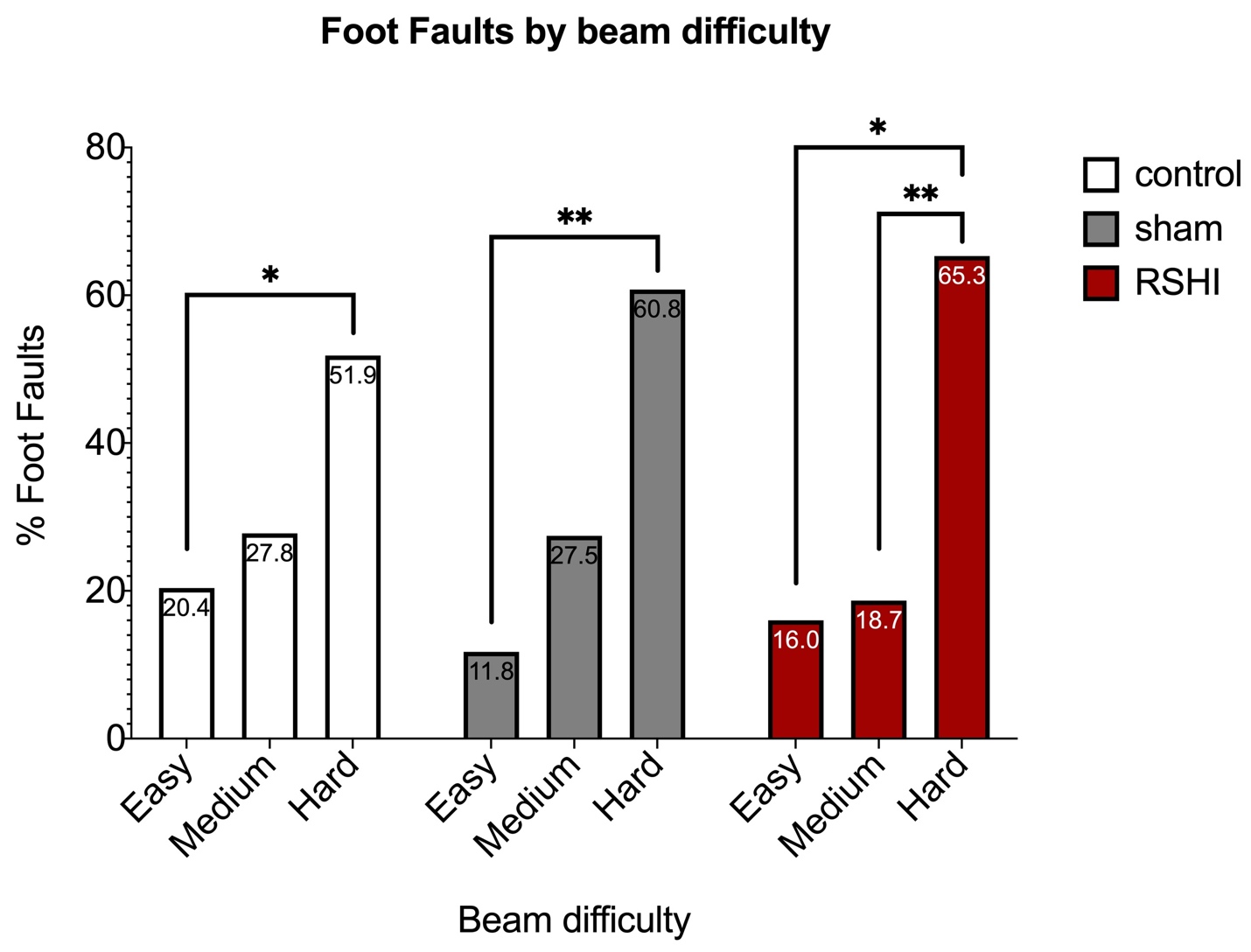


**Supplementary Figure 18.** Foot faults increase as beam narrows

For all groups, the majority of foot faults (52–65%) were made on the hardest (i.e., narrowest) section of the beam (interval 3) (RSHI *P* = 0.0009; sham *P* = 0.001; controls *P* = 0.08) with a trend towards group differences (*P* = 0.087). After stratification by sex, group differences in interval 3 were borderline significant for males (*P* = 0.06), but not females (*P* = 0.55). Male RSHI animals had numerically more foot faults in interval 3 than female RSHI animals (*P* = 0.11).


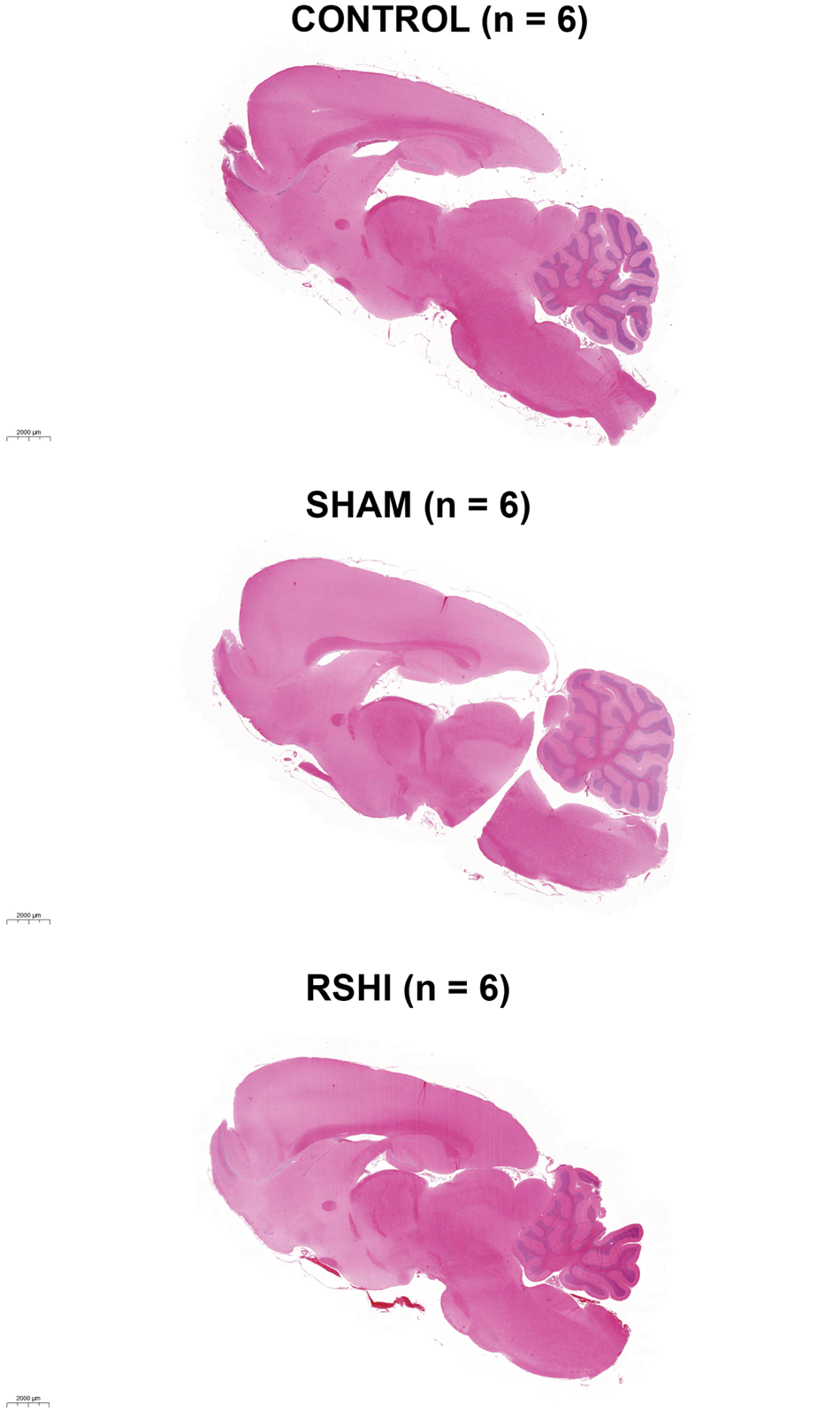


Supplementary Figure 19. H&E-stained sections show no gross morphological abnormalities

Review of sections by a comparative pathologist (Dr. Amanda Beck) revealed no abnormalities including evidence of prior hemorrhage or contusion. A common artifactual finding in all brain sections was basophilic or dark staining neurons, a common artifact seen in non-perfused rodent brains.


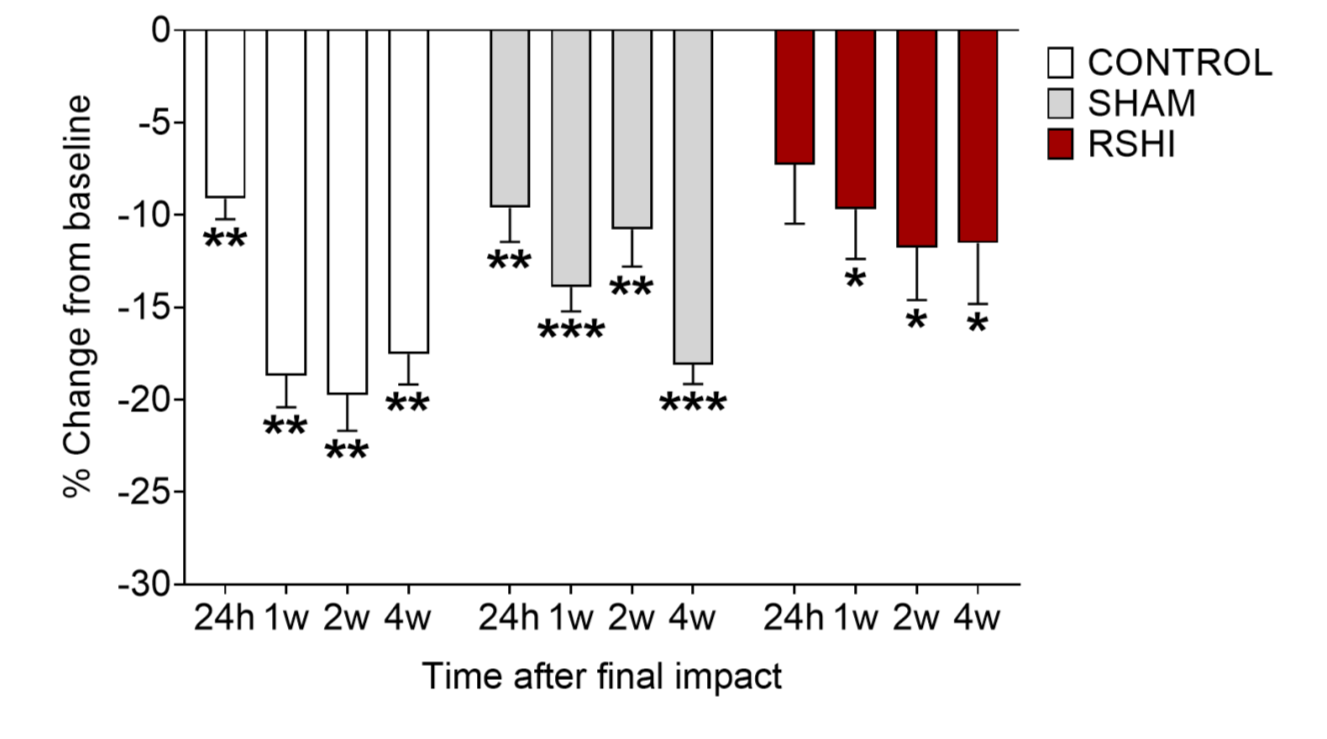


**Supplementary Figure 20.** Longitudinal radial diffusivity of the corpus callosum following the final impact.

The corpus callosum shows significant age-dependent development, expressed by decreases in RD in all groups, a possible sign of myelination, but to a lesser degree in RSHI animals. The vertical axis represents percentage change from pre-injury baseline MRI. The horizontal axis represents the amount of time after completion of the impact protocol: 24h = 24 hours; 1w = 1 week; 2w = 2 weeks; 4w = 4 weeks. Control *n* = 6, Sham *n* = 8, RSHI *n* = 8. All data are presented as mean ± standard error of the mean. **P* < 0.05, ***P* < 0.01, ****P* < 0.001.

**References**

1. Smith SM, Johansen-Berg H, Jenkinson M, et al. Acquisition and voxelwise analysis of multi-subject diffusion data with tract-based spatial statistics. *Nat Protoc*. 2007;2(3):499-503. doi:10.1038/nprot.2007.45

2. Hulkower MB, Poliak DB, Rosenbaum SB, Zimmerman ME, Lipton ML. A decade of DTI in traumatic brain injury: 10 years and 100 articles later. *AJNR Am J Neuroradiol*. Nov-Dec 2013;34(11):2064-74. doi:10.3174/ajnr.A3395

3. Hoogenboom WS, Rubin TG, Ye K, et al. Diffusion Tensor Imaging of the Evolving Response to Mild Traumatic Brain Injury in Rats. *J Exp Neurosci*. 2019;13:1-12. doi:10.1177/1179069519858627

4. Paxinos G, Watson CR. *The Rat Brain in Stereotaxic Coordinates*. Second Edition ed. Academic Press Inc.; 1986:264.

5. Boehm SL, Schafer GL, Phillips TJ, Browman KE, Crabbe JC. Sensitivity to ethanol-induced motor incoordination in 5-HT(1B) receptor null mutant mice is task-dependent: implications for behavioral assessment of genetically altered mice. *Behav Neurosci*. Apr 2000;114(2):401-9.

6. Stanley JL, Lincoln RJ, Brown TA, McDonald LM, Dawson GR, Reynolds DS. The mouse beam walking assay offers improved sensitivity over the mouse rotarod in determining motor coordination deficits induced by benzodiazepines. *J Psychopharmacol*. May 2005;19(3):221-7. doi:10.1177/0269881105051524

7. Zhao CS, Puurunen K, Schallert T, Sivenius J, Jolkkonen J. Behavioral effects of photothrombotic ischemic cortical injury in aged rats treated with the sedative-hypnotic GABAergic drug zopiclone. *Behav Brain Res*. May 2005;160(2):260-6. doi:10.1016/j.bbr.2004.12.007

8. Zhao CS, Puurunen K, Schallert T, Sivenius J, Jolkkonen J. Effect of cholinergic medication, before and after focal photothrombotic ischemic cortical injury, on histological and functional outcome in aged and young adult rats. *Behav Brain Res*. Jan 2005;156(1):85-94. doi:10.1016/j.bbr.2004.05.011

9. Lynn DA, Brown GR. The ontogeny of exploratory behavior in male and female adolescent rats (Rattus norvegicus). *Dev Psychobiol*. Sep 2009;51(6):513-20. doi:10.1002/dev.20386

10. Seibenhener ML, Wooten MC. Use of the Open Field Maze to measure locomotor and anxiety-like behavior in mice. *J Vis Exp*. Feb 2015;(96):e52434. doi:10.3791/52434

11. Walf AA, Frye CA. The use of the elevated plus maze as an assay of anxiety-related behavior in rodents. *Nat Protoc*. 2007;2(2):322-8. doi:10.1038/nprot.2007.44

12. Otvos L, Feiner L, Lang E, Szendrei GI, Goedert M, Lee VM. Monoclonal antibody PHF-1 recognizes tau protein phosphorylated at serine residues 396 and 404. *J Neurosci Res*. Dec 1994;39(6):669-73. doi:10.1002/jnr.490390607

13. Greenberg SG, Davies P, Schein JD, Binder LI. Hydrofluoric acid-treated tau PHF proteins display the same biochemical properties as normal tau. *J Biol Chem*. Jan 1992;267(1):564-9.

14. Wu TC, Wilde EA, Bigler ED, et al. Longitudinal changes in the corpus callosum following pediatric traumatic brain injury. *Dev Neurosci*. 2010;32(5-6):361-73. doi:10.1159/000317058
